# Supplementary material for: An Extended Reservoir of Class-D Beta-Lactamases in Non-Clinical Bacterial Strains
Source: Microbiol Spectr. 2022 Mar 21;10(2):e00315-22. doi: 10.1128/spectrum.00315-22 (PMC9045261; doi:10.1128/spectrum.00315-22)

Table S1. **Details of the 64 phylogenetic clusters.** The retained clustering (x set at 0.20 and inflation at 1.5; see Materials and Methods) had a computed entropy of 0.762 and a score of 0.52. BLAST-based annotation (identity threshold of 90%) of the clusters was derived from sub-family annotations (if any, unless the protein name is used) of the class-D beta-lactamases from the Beta-lactamase Database (BLDB).

Cluster	# seqs (representative)	# seqs (unique)	# seqs (all)	# DBL-homologs (unique)	# BlaR-homologs (unique)	Taxonomy	BLDB sub-family	Active site logo
cluster1	4	5	5	5	0	Oligoflexia		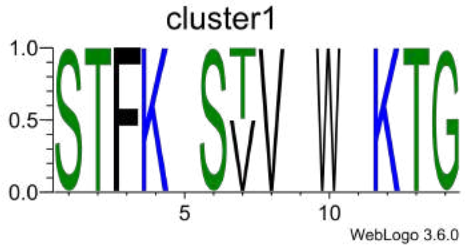
cluster2	16	31	860	31	0	Betaproteobacteria, Deltaproteobacteria, Gammaproteobacteria	OXA1	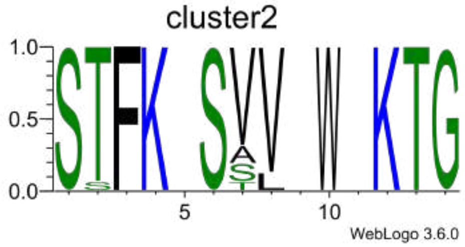
cluster3	46	79	767	79	0	Chlamydiae, Alphaproteobacteria, Deltaproteobacteria, Gammaproteobacteria	OXA29	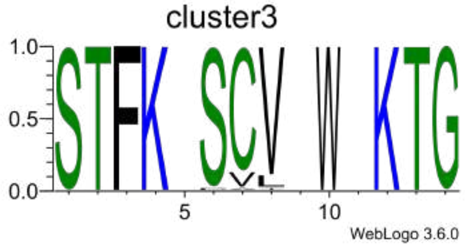

cluster4	1	1	2	1	0	Alphaproteobacteria		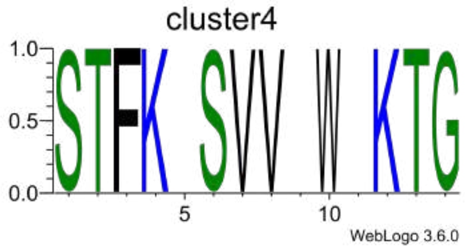
cluster5	2	3	3	3	0	Alphaproteobacteria		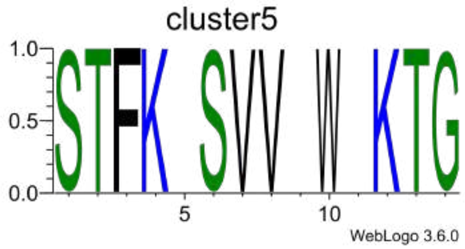
cluster6	16	98	164	98	0	Gammaproteobacteria	OXA12	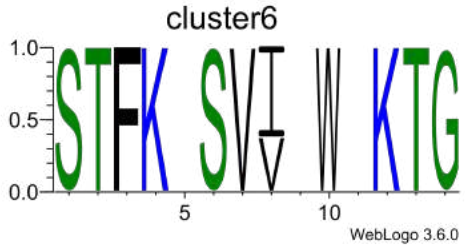
cluster7	98	170	719	170	0	Planctomycetes, Verrucomicrobia, Alphaproteobacteria, Gammaproteobacteria	OXA9	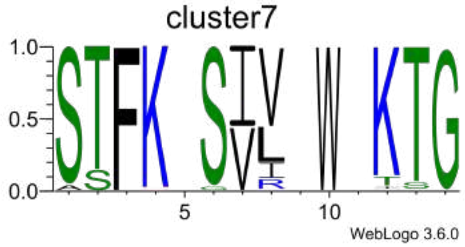

cluster8	195	561	1768	546	15	Alphaproteobacteria, Betaproteobacteria, Gammaproteobacteria	OXA114a, OXA22, OXA243, OXA258, OXA42, OXA457	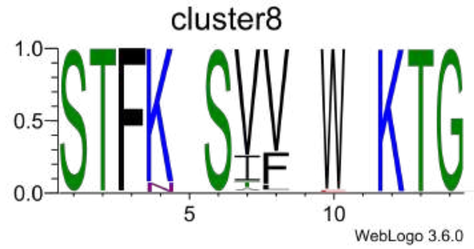
cluster9	1	1	1	1	0	Deltaproteobacteria		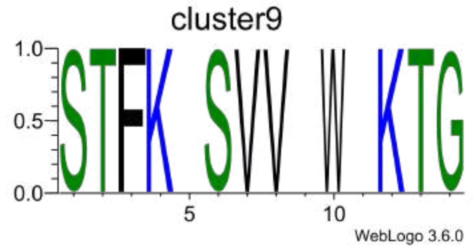
cluster10	1	1	1	1	0	Firmicutes		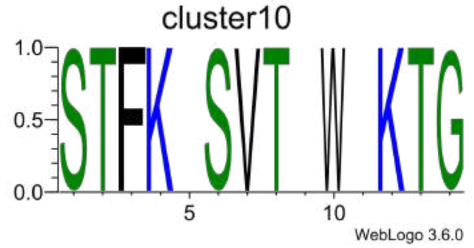
cluster11	4	6	6	6	0	Deltaproteobacteria		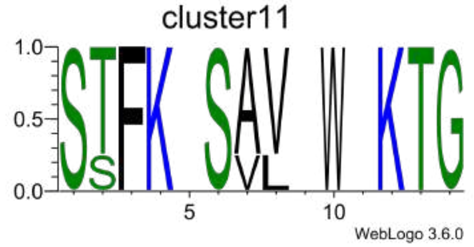

cluster12	8	12	16	12	0	Gammaproteobacteria		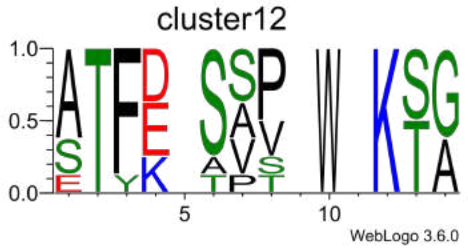
cluster13	1	1	1	1	0	Spirochaetes		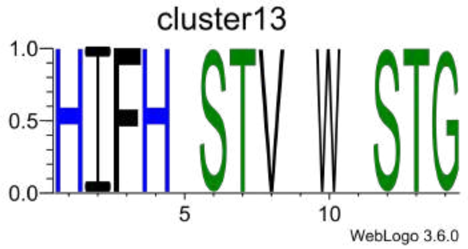
cluster14	11	72	415	72	0	Spirochaetes		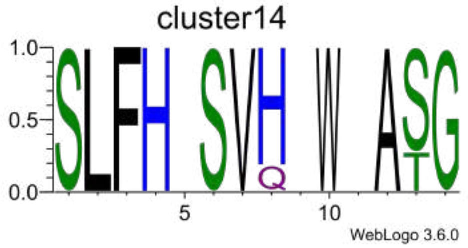
cluster15	207	396	1306	28	368	Firmicutes, Fusobacteria, Deltaproteobacteria	BLAR1	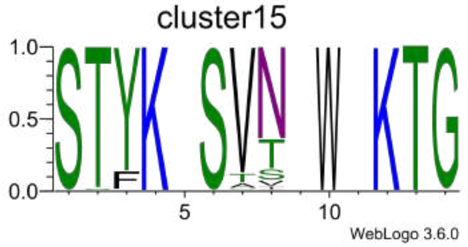

cluster16	4	5	13	0	5	Firmicutes		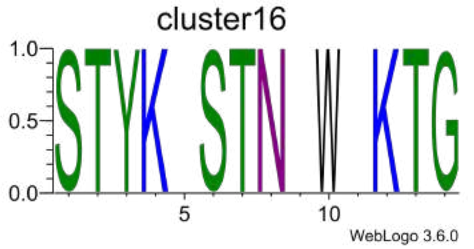
cluster17	1	1	1	0	1	Firmicutes		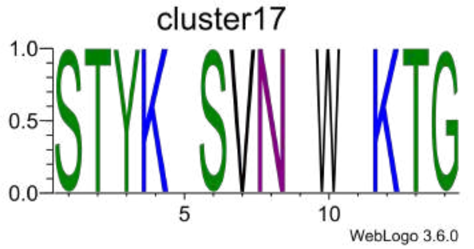
cluster18	11	478	9214	18	460	Firmicutes	BLAR1	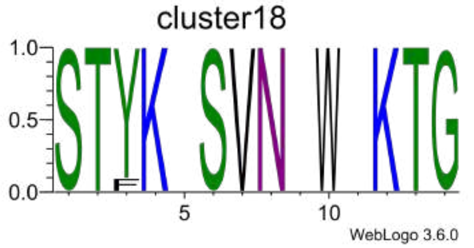
cluster19	83	209	374	209	0	Firmicutes	BAC1, BAD1, BAT1, BED1, BEN1, BOC1, BPU1, BSD1, BSU1, YBXI	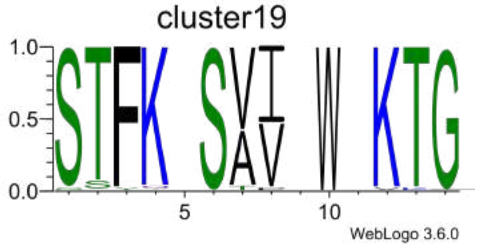

cluster20	9	9	9	9	0	Chlorobi, Verrucomicrobia, Deltaproteobacteria		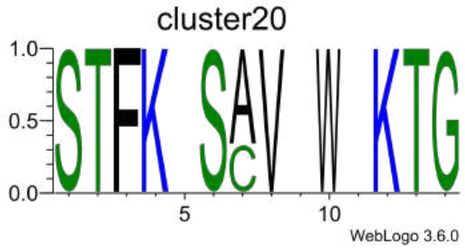
cluster21	38	87	956	87	0	Firmicutes	CDD	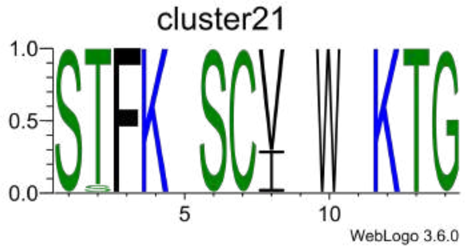
cluster22	24	25	27	25	0	Bacteroidetes, Chloroflexi, Betaproteobacteria, Deltaproteobacteria, Gammaproteobacteria		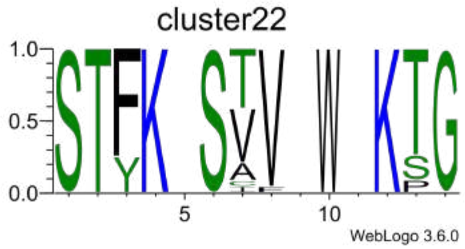
cluster23	23	24	25	24	0	Bacteroidetes		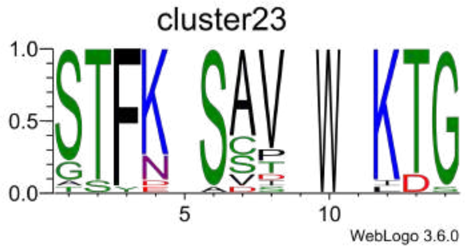

cluster24	1	1	1	1	0	Bacteroidetes		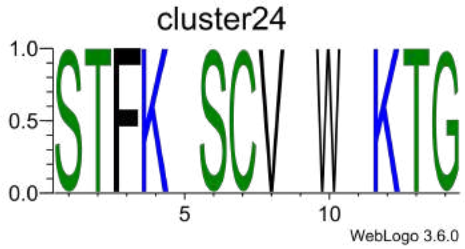
cluster25	1	1	1	1	0	Bacteroidetes		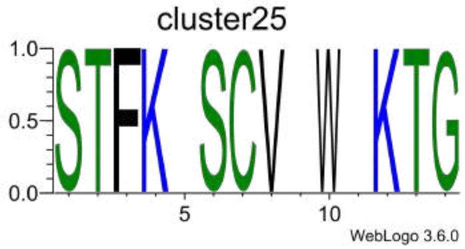
cluster26	6	6	6	6	0	Bacteroidetes		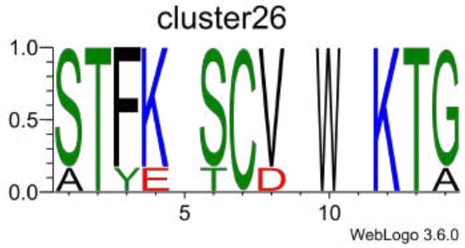
cluster27	44	48	82	48	0	Bacteroidetes	OXA347	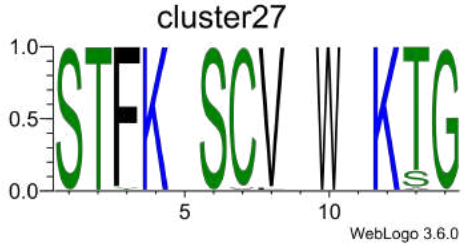

cluster28	55	62	70	62	0	Bacteroidetes, Fusobacteria, Balneolaeota		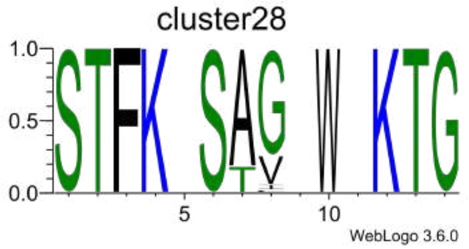
cluster29	1	1	1	1	0	Bacteroidetes		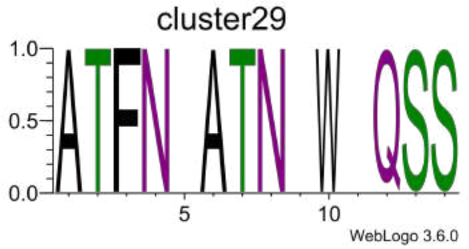
cluster30	53	60	81	60	0	Cyanobacteria, Bacteroidetes, Spirochaetes, Verrucomicrobia, Gammaproteobacteria		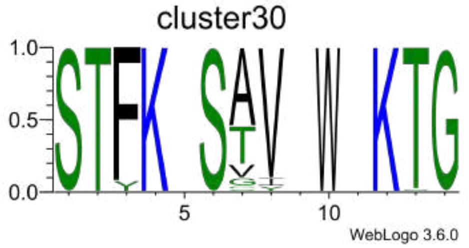
cluster31	3	3	4	3	0	Bacteroidetes, Deltaproteobacteria		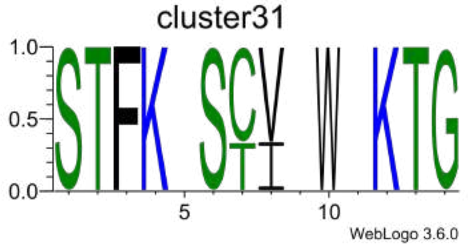

cluster32	4	4	4	4	0	Gammaproteobacteria		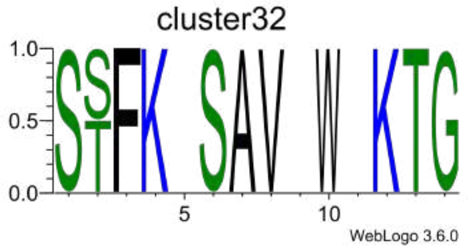
cluster33	4	4	4	4	0	Alphaproteobacteria		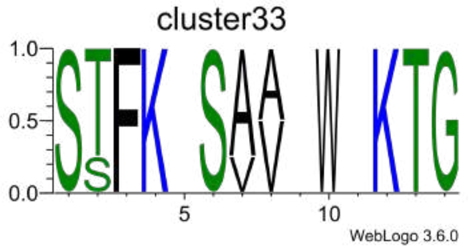
cluster34	26	36	39	36	0	Firmicutes, Alphaproteobacteria, Deltaproteobacteria, Epsilonproteobacteria, Gammaproteobacteria	OXA464	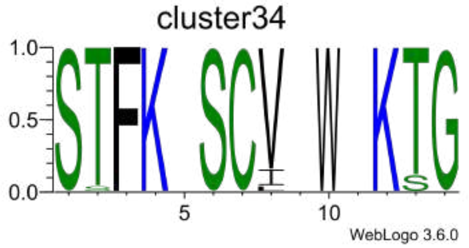
cluster35	1	1	2	1	0	Gammaproteobacteria		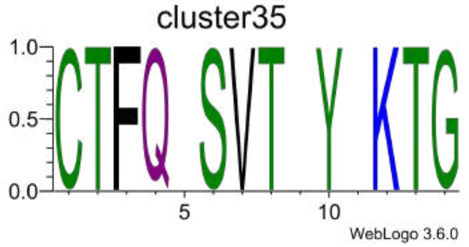

cluster36	2	2	2	2	0	Gammaproteobacteria		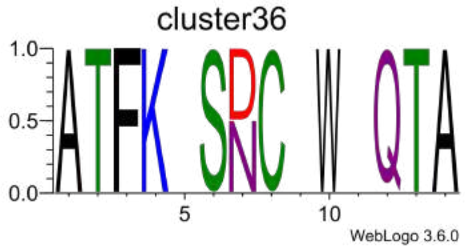
cluster37	29	48	55	48	0	Chlorobi, Betaproteobacteria, Gammaproteobacteria	LCRNPS	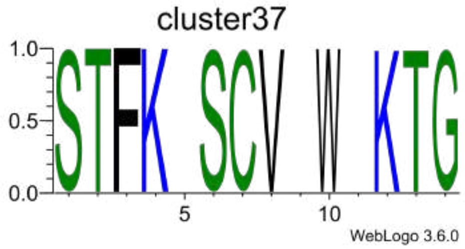
cluster38	2	2	2	2	0	Betaproteobacteria		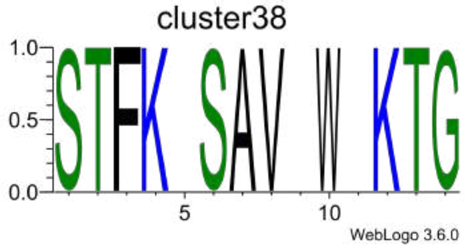
cluster39	16	16	18	16	0	Alphaproteobacteria		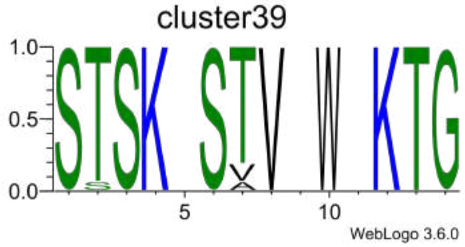

cluster40	43	81	217	81	0	Cyanobacteria, Alphaproteobacteria, Betaproteobacteria, Gammaproteobacteria	OXA2, OXA20, OXA46	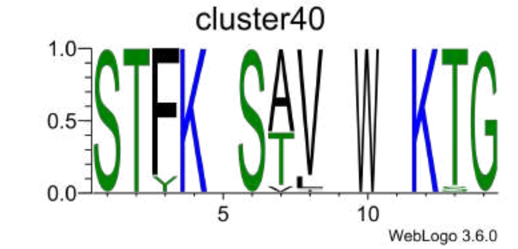
cluster41	6	7	7	7	0	Gammaproteobacteria		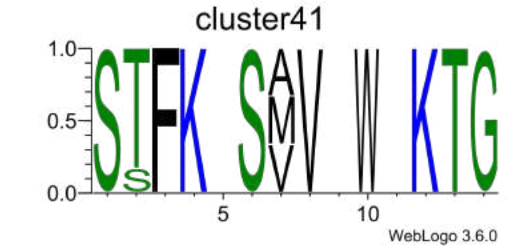
cluster42	11	15	23	15	0	Gammaproteobacteria		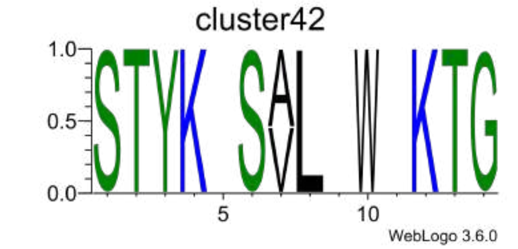
cluster43	19	57	263	57	0	Betaproteobacteria, Gammaproteobacteria	OXA10, OXA48, OXA5, OXA548, OXA55	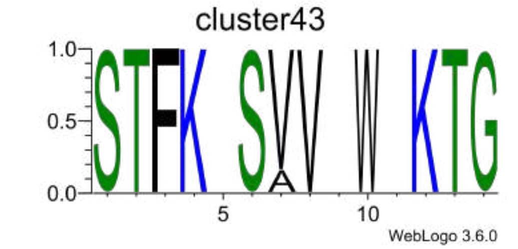

cluster44	55	106	141	106	0	Alphaproteobacteria		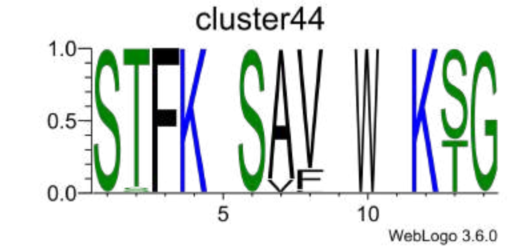
cluster45	1	1	1	1	0	Alphaproteobacteria		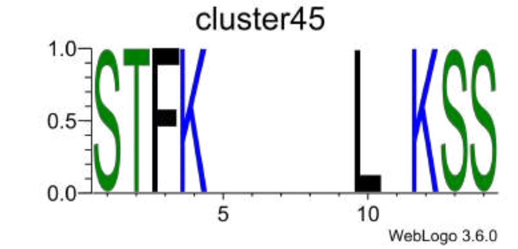
cluster46	1	1	1	1	0	Betaproteobacteria		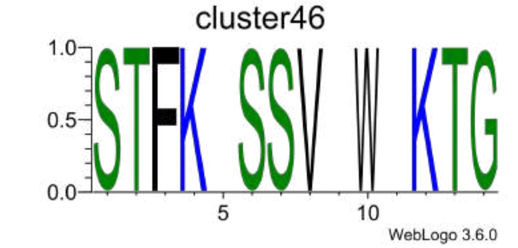
cluster47	3	3	3	3	0	Betaproteobacteria, Gammaproteobacteria		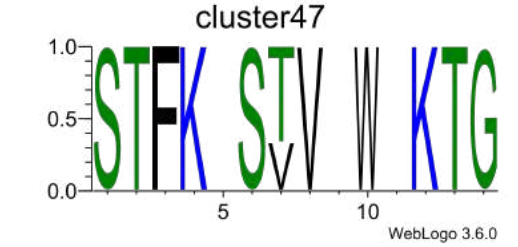

cluster48	1	1	1	1	0	Gammaproteobacteria		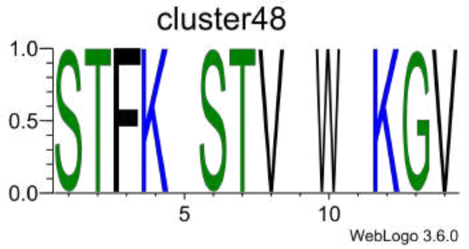
cluster49	1	1	1	1	0	Bacteroidetes		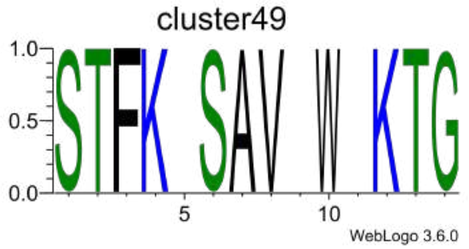
cluster50	2	2	2	2	0	Deltaproteobacteria		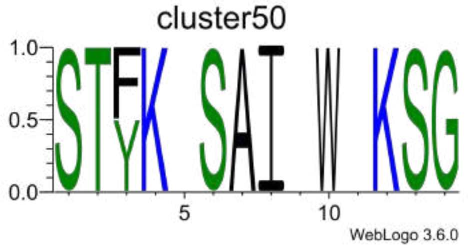
cluster51	2	2	2	2	0	Alphaproteobacteria		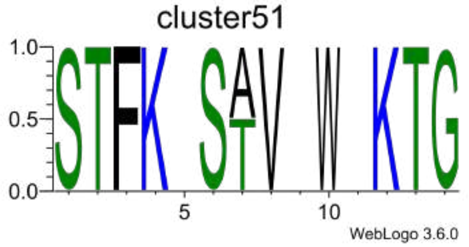

cluster52	1	1	1	1	0	Deltaproteobacteria		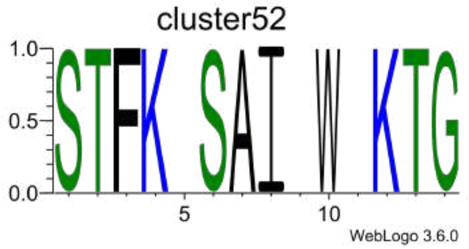
cluster53	1	1	1	1	0	Deltaproteobacteria		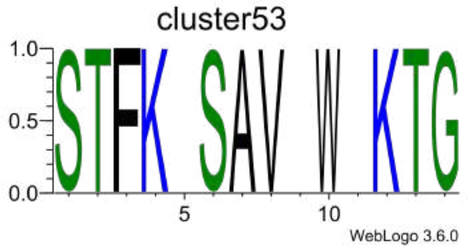
cluster54	2	2	2	2	0	Alphaproteobacteria		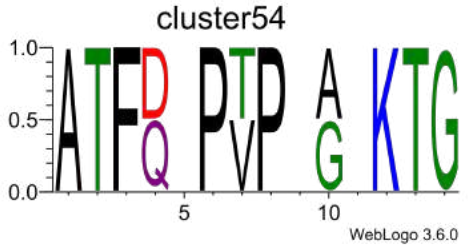
cluster55	1	1	1	1	0	Alphaproteobacteria		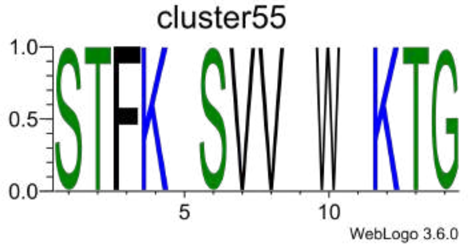

cluster56	5	5	6	5	0	Deltaproteobacteria		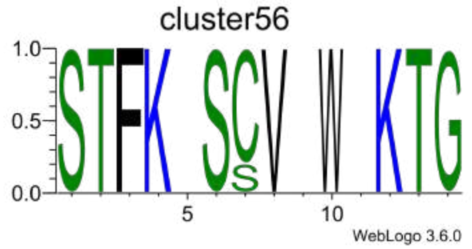
cluster57	9	15	21	15	0	Betaproteobacteria		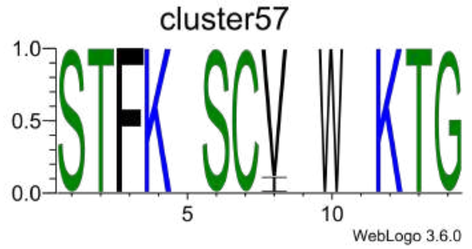
cluster58	12	17	22	17	0	Betaproteobacteria	OXA153, OXA154, OXA156, OXA157, OXA62	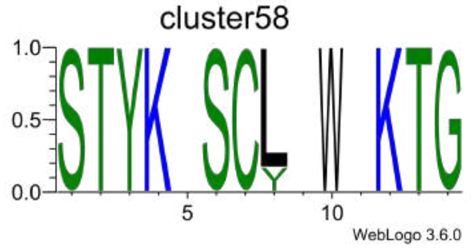
cluster59	1	1	1	1	0	Alphaproteobacteria		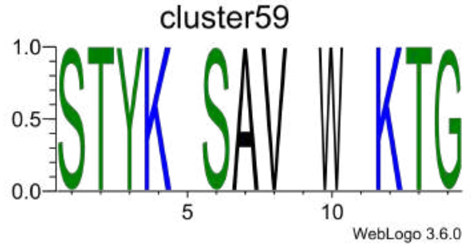

cluster60	66	313	3472	313	0	Firmicutes, Alphaproteobacteria, Betaproteobacteria, Gammaproteobacteria	OXA134, OXA143, OXA211, OXA213, OXA214, OXA228, OXA23, OXA24, OXA266, OXA274, OXA279, OXA286, OXA294, OXA296, OXA299, OXA308, OXA51, OXA58, OXA665	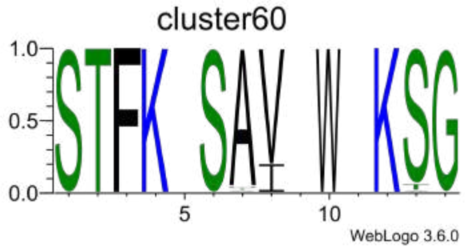
cluster61	93	195	2361	195	0	Actinobacteria, Verrucomicrobia, Cyanobacteria, Fusobacteria, Alphaproteobacteria, Betaproteobacteria, Deltaproteobacteria, Epsilonproteobacteria, Gammaproteobacteria	OXA50, OXA60	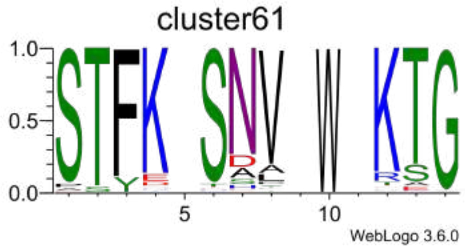
cluster62	1	1	1	1	0	Epsilonproteobacteria		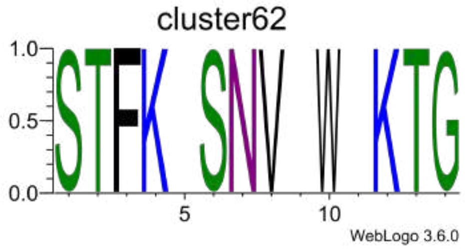

cluster63	1	1	1	1	0	Gammaproteobacteria		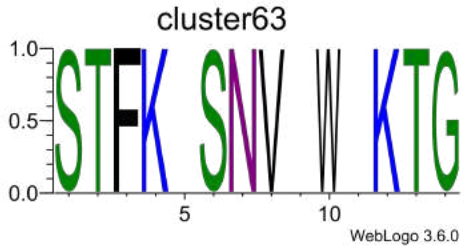
cluster64	23	110	1339	110	0	Spirochaetes, Fusobacteria, Epsilonproteobacteria	OXA184, OXA493, OXA576, OXA61, OXA63, OXA85	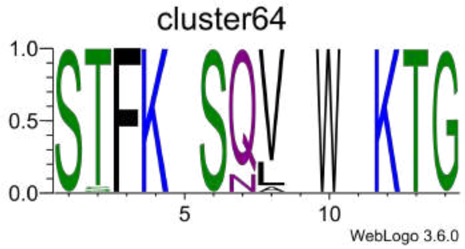

Table S2. **Details of the ten sequences selected for production.**

Cluster	Protein accession	Len. (AA)	Signal peptide	# TM	Encoding molecule	Strain status	Organism
cluster14	WP_003003775.1 (OXAVL01)	278	Lipo	0	chromosome	non-clinical	Leptospira weilii serovar Ranarum str. ICFT (GCF_000332415.1)
cluster22	WP_075071939.1 (OXAVL02)	289	Lipo	0	chromosome	non-clinical	Longilinea arvoryzae (GCF_001050235.1)
cluster23	WP_039139369.1 (OXAVL03)	272	Lipo	0	chromosome	non-clinical	Flavihumibacter solisilvae (GCF_000814475.1)
cluster28	WP_012859784.1 (OXAVL04)	264	Sec	0	chromosome	unclassified	Sebaldella termitidis ATCC 333866 (GCF_000024405.1)
cluster30	WP_017307273.1 (OXAVL05)	283	Sec	0	unclassified	non-clinical	Spirulina subsalsa PCC 9445 (GCF_000314005.1)
cluster39	WP_051601260.1 (OXAVL06)	274	Lipo	0	chromosome	non-clinical	Hyphomonas beringensis (GCF_000682755.1)
cluster41	WP_011041344.1 (OXAVL07)	303	Sec	0	chromosome	unclassified	Colwellia psychrerythraea 34H (GCF_000012325.1)
cluster42	WP_023398243.1 (OXAVL08)	262	Sec	0	chromosome	unclassified	Pseudoalteromonas luteoviolacea NCIMB 1944 (GCF_001625565.1)
cluster44	WP_012045014.1 (OXAVL09)	272	Tat	0	unclassified	unclassified	Bradyrhizobium sp. BTAi1 (GCF_000015165.1)
cluster57	WP_042878825.1 (OXAVL10)	278	Sec	0	unclassified	non-clinical	Cupriavidus necator A5-1 (GCF_000744095.1)

Table S3. **Quantile values of the patristic distances within each cluster.** The patristic distance is the sum of the branch lengths connecting two leaves in the phylogenetic tree. Here all intra-cluster pairwise combinations are considered.

Cluster	# seqs	Min	P25	Median	P75	Max
cluster1	4	0.00969	0.2059525	0.676575	0.7019525	0.71252
cluster2	16	0	0.415955	0.5568	0.7226125	1.0108
cluster3	46	0.01466	0.361375	0.81088	1.026605	1.67419
cluster5	2	0.27086	0.27086	0.27086	0.27086	0.27086
cluster6	16	0.01953	0.15301	0.21419	0.31377	0.54345
cluster7	98	0.01905	0.69301	0.86071	1.03713	1.92185
cluster8	195	0	0.770885	0.93889	1.110865	1.97655
cluster11	4	0.08396	0.30977	0.56715	0.6112075	0.69586
cluster12	8	0.04397	0.6758725	0.839215	0.95245	1.48239
cluster14	11	0.044	0.19852	0.24713	0.294545	0.41045
cluster15	207	0	0.92162	1.06751	1.22639	2.13709
cluster16	4	0.03267	0.2215675	0.40052	0.59001	0.60036
cluster18	11	0.00888	0.086785	0.90216	0.959735	1.07516
cluster19	83	0.00917	0.545395	0.72186	0.847135	1.48553
cluster20	9	0.04945	0.398155	0.715475	0.8981125	1.11107
cluster21	38	0.02332	0.77539	0.95729	1.162505	1.50658
cluster22	24	0.04456	0.67553	1.034565	1.400815	1.71683
cluster23	23	0.04849	0.5105	0.62438	0.78451	1.37281
cluster26	6	0.15873	0.49242	0.72263	0.83551	1.06572
cluster27	44	0.02671	0.5849225	0.738465	0.9223375	1.36627
cluster28	55	0.03899	0.62546	0.82497	1.00318	1.7674
cluster30	53	0.02332	0.4809275	0.62634	1.00227	1.61624
cluster31	3	0.30642	0.616195	0.92597	0.98358	1.04119
cluster32	4	0.25138	0.26632	0.30462	0.405275	0.441
cluster33	4	0.22863	0.6938975	0.77468	1.0105775	1.06262
cluster34	26	0.07511	0.80561	0.96124	1.10099	1.44135
cluster36	2	0.05568	0.05568	0.05568	0.05568	0.05568
cluster37	29	0.03376	0.6354725	0.80797	0.9243375	1.29984
cluster38	2	0.31643	0.31643	0.31643	0.31643	0.31643
cluster39	16	0.05838	0.419635	0.53467	0.958935	1.13721
cluster40	43	0.02916	0.463685	0.58767	0.694815	1.00944
cluster41	6	0.08239	0.678715	0.81042	0.91188	1.02917
cluster42	11	0.06239	0.267915	0.39539	0.66776	0.75012
cluster43	19	0.02369	0.57327	0.70553	0.865345	1.32908
cluster44	55	0.0192	0.20912	0.31989	0.40854	0.96414

cluster47	3	0.22092	0.556305	0.89169	0.93107	0.97045
cluster50	2	0.29347	0.29347	0.29347	0.29347	0.29347
cluster51	2	0.58105	0.58105	0.58105	0.58105	0.58105
cluster54	2	0.58559	0.58559	0.58559	0.58559	0.58559
cluster56	5	0.07076	0.40092	0.722035	0.7739	0.82261
cluster57	9	0.02085	0.0984025	0.298545	0.443115	0.53549
cluster58	12	0.01854	0.105465	0.14054	0.17294	0.23443
cluster60	66	0.01423	0.52437	0.61548	0.78495	1.57714
cluster61	93	0.0097	0.85578	1.085395	1.3369775	2.30433
cluster64	23	0	0.33179	0.56669	0.93299	1.25124

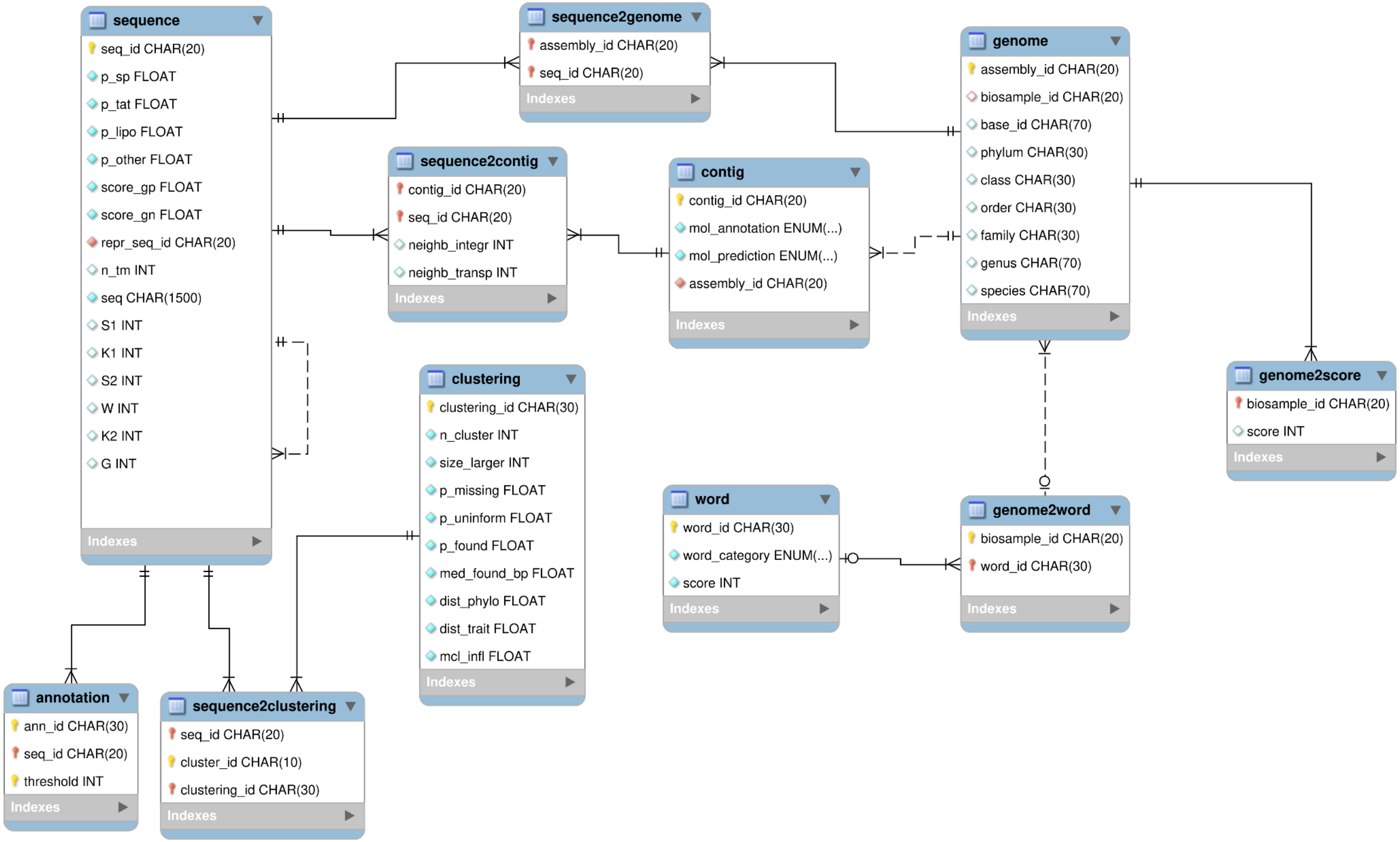

Figure S1. **Schematic representation of the SQL database.**

Figure S2. **SEC and SEC-MALS analysis performed on the purified OXAVL06.** (**A**) Size Exclusion Chromatography (SEC) analysis of the purified OXAVL06. (**B-D**) Determination of the multimeric state of OXAVL06 (peaks 1, 2 and 3) by SEC-MALS (Multi-Angle Light Scattering) analysis.

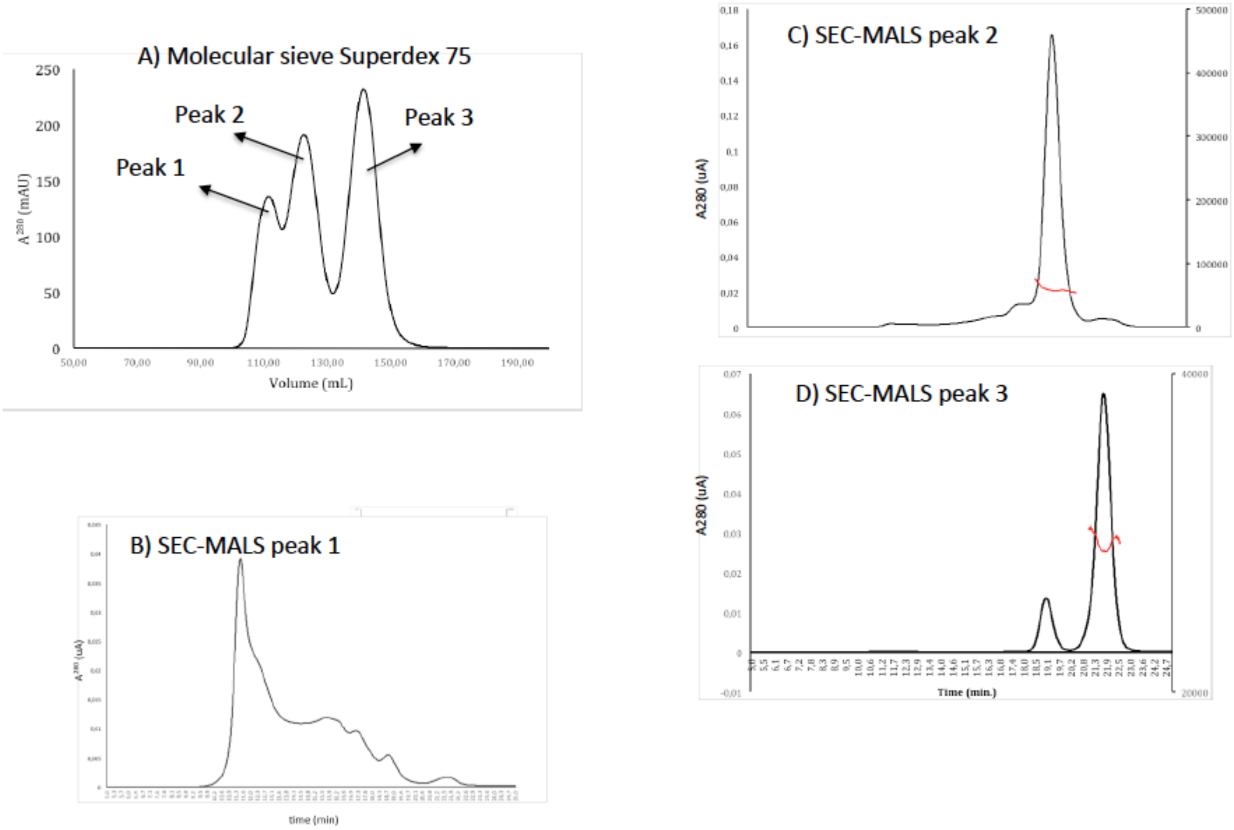

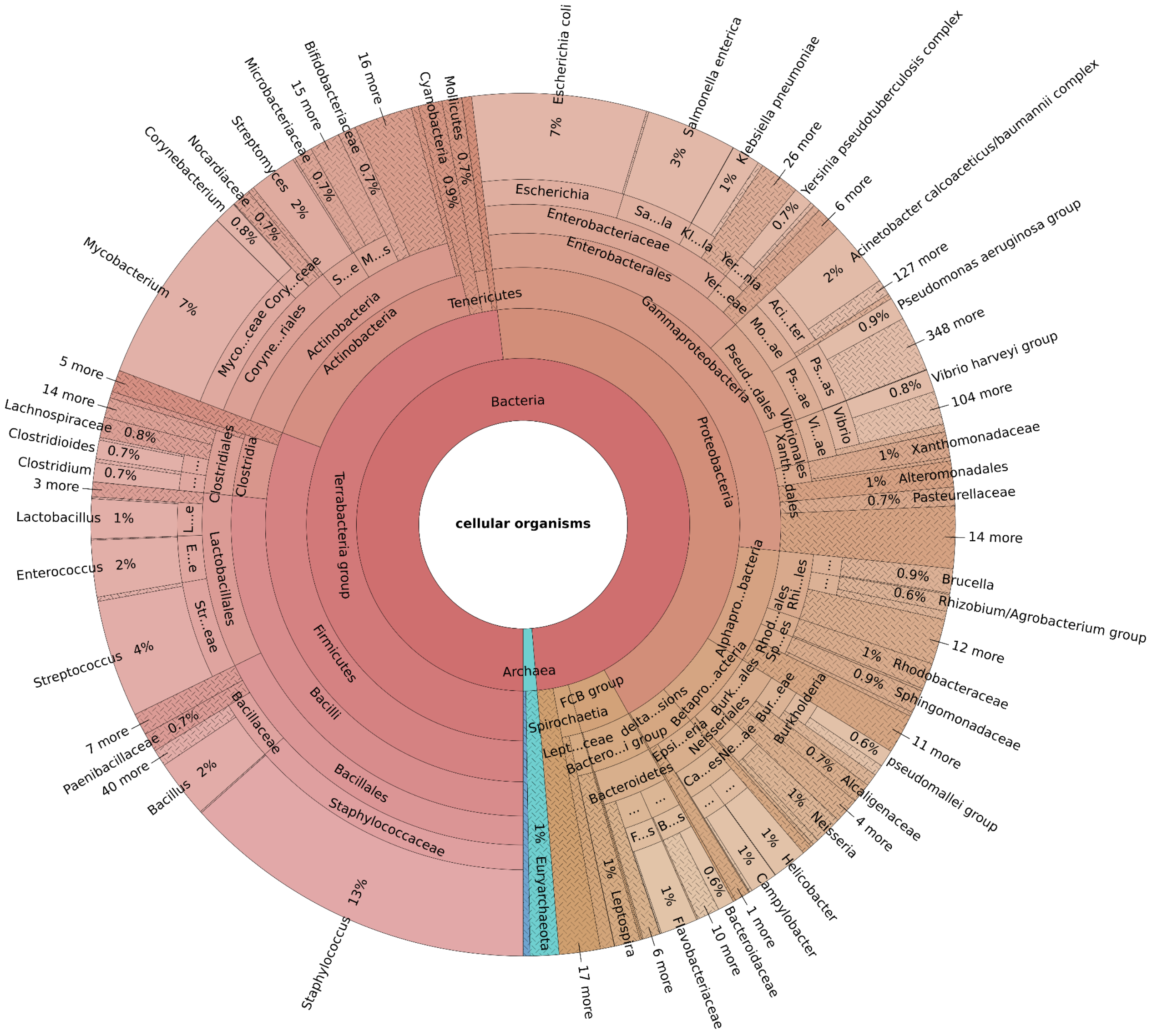

Figure S3. **Krona chart of the taxonomic diversity of the local NCBI RefSeq database.**

Supplemental Data 1

InterProScan raw results

GCF_001758545.1|WP_071849985.1619	ProSitePatterns	PS00337	Beta-lactamase class-D active site.	49	59	-
GCF_001758545.1|WP_071849985.1619	Pfam	PF00144	Beta-lactamase	265	613	5.0E-65
GCF_001758545.1|WP_071849985.1619	Pfam	PF00905	Penicillin binding protein transpeptidase domain	39	230	8.2E-30
GCF_001758545.1|WP_071849985.1619	SUPERFAMILY	SSF56601		262	614	1.11E-84
GCF_001758545.1|WP_071849985.1619	ProSitePatterns	PS00336	Beta-lactamase class-C active site.	313	320	-
GCF_001758545.1|WP_071849985.1619	SUPERFAMILY	SSF56601		26	235	6.91E-27
GCF_001758545.1|WP_071849985.1619	Gene3D	G3DSA:3.40.710.10		255	617	3.4E-116
GCF_001758545.1|WP_071849985.1619	Gene3D	G3DSA:3.40.710.10		24	254	3.3E-74
GCF_001423125.1|WP_056159201.1633	Pfam	PF00905	Penicillin binding protein transpeptidase domain	407	619	4.5E-33
GCF_001423125.1|WP_056159201.1633	Gene3D	G3DSA:3.40.710.10		391	632	2.4E-71
GCF_001423125.1|WP_056159201.1633	Pfam	PF05569	BlaR1 peptidase M56	27	291	6.5E-27
GCF_001423125.1|WP_056159201.1633	ProSitePatterns	PS00337	Beta-lactamase class-D active site.	418	428	-
GCF_001423125.1|WP_056159201.1633	CDD	cd07341	M56_BlaR1_MecR1_like	134	324	6.12734E-30
GCF_001423125.1|WP_056159201.1633	SUPERFAMILY	SSF56601		384	624	3.42E-32
GCF_900129765.1|WP_073220251.1573	Pfam	PF00144	Beta-lactamase	220	562	9.1E-68
GCF_900129765.1|WP_073220251.1573	SUPERFAMILY	SSF56601		6	203	1.04E-25
GCF_900129765.1|WP_073220251.1573	ProSitePatterns	PS00337	Beta-lactamase class-D active site.	12	22	-
GCF_900129765.1|WP_073220251.1573	ProSitePatterns	PS00336	Beta-lactamase class-C active site.	268	275	-
GCF_900129765.1|WP_073220251.1573	Gene3D	G3DSA:3.40.710.10		1	217	4.1E-73
GCF_900129765.1|WP_073220251.1573	Pfam	PF00905	Penicillin binding protein transpeptidase domain	4	191	4.1E-31
GCF_900129765.1|WP_073220251.1573	Gene3D	G3DSA:3.40.710.10		218	571	2.8E-119
GCF_900129765.1|WP_073220251.1573	SUPERFAMILY	SSF56601		219	568	1.22E-86
GCF_000344615.1|WP_017879576.1613	Pfam	PF05569	BlaR1 peptidase M56	13	306	1.4E-28
GCF_000344615.1|WP_017879576.1613	SUPERFAMILY	SSF56601		372	596	4.13E-30

GCF_000344615.1|WP_017879576.1613	Pfam	PF00905	Penicillin binding protein transpeptidase domain	387	599	2.0E-41
GCF_000344615.1|WP_017879576.1613	Gene3D	G3DSA:3.40.710.10		369	613	2.7E-82
GCF_000344615.1|WP_017879576.1613	CDD	cd07341	M56_BlaR1_MecR1_like	131	306	1.94503E-32
GCF_000315425.1|WP_005669363.1571	Pfam	PF05569	BlaR1 peptidase M56	156	289	1.2E-26
GCF_000315425.1|WP_005669363.1571	SUPERFAMILY	SSF56601		328	557	6.91E-29
GCF_000315425.1|WP_005669363.1571	CDD	cd07341	M56_BlaR1_MecR1_like	119	245	1.62457E-28
GCF_000315425.1|WP_005669363.1571	Pfam	PF00905	Penicillin binding protein transpeptidase domain	354	557	1.5E-29
GCF_000315425.1|WP_005669363.1571	Gene3D	G3DSA:3.40.710.10		334	571	1.9E-72
GCF_001191005.1|WP_050407176.1598	Pfam	PF00905	Penicillin binding protein transpeptidase domain	373	584	6.9E-36
GCF_001191005.1|WP_050407176.1598	Gene3D	G3DSA:3.40.710.10		354	598	1.3E-77
GCF_001191005.1|WP_050407176.1598	SUPERFAMILY	SSF56601		354	575	6.87E-30
GCF_001191005.1|WP_050407176.1598	Pfam	PF05569	BlaR1 peptidase M56	11	307	2.8E-34
GCF_900129765.1|WP_073218605.1598	SUPERFAMILY	SSF56601		357	586	5.27E-32
GCF_900129765.1|WP_073218605.1598	Gene3D	G3DSA:3.40.710.10		354	598	1.8E-80
GCF_900129765.1|WP_073218605.1598	Pfam	PF05569	BlaR1 peptidase M56	117	285	1.8E-26
GCF_900129765.1|WP_073218605.1598	CDD	cd07341	M56_BlaR1_MecR1_like	169	306	4.50559E-20
GCF_900129765.1|WP_073218605.1598	Pfam	PF00905	Penicillin binding protein transpeptidase domain	366	585	2.4E-40
GCF_001425045.1|WP_057157847.1599	Pfam	PF05569	BlaR1 peptidase M56	19	306	8.6E-35
GCF_001425045.1|WP_057157847.1599	CDD	cd07341	M56_BlaR1_MecR1_like	134	307	4.81022E-28
GCF_001425045.1|WP_057157847.1599	MobiDBLite	mobidb-lite	consensus disorder prediction	83	106	-
GCF_001425045.1|WP_057157847.1599	SUPERFAMILY	SSF56601		356	585	1.82E-32
GCF_001425045.1|WP_057157847.1599	Pfam	PF00905	Penicillin binding protein transpeptidase domain	372	584	7.3E-38
GCF_001425045.1|WP_057157847.1599	Gene3D	G3DSA:3.40.710.10		356	599	5.0E-81
GCF_001758545.1|WP_071849986.1604	Pfam	PF00905	Penicillin binding protein transpeptidase domain	376	591	6.7E-35
GCF_001758545.1|WP_071849986.1604	Gene3D	G3DSA:3.40.710.10		360	604	3.0E-77
GCF_001758545.1|WP_071849986.1604	CDD	cd07341	M56_BlaR1_MecR1_like	123	299	1.76121E-32
GCF_001758545.1|WP_071849986.1604	Pfam	PF05569	BlaR1 peptidase M56	12	288	2.9E-32
GCF_001758545.1|WP_071849986.1604	SUPERFAMILY	SSF56601		363	587	8.54E-31
GCF_001425265.1|WP_056397199.1609	SUPERFAMILY	SSF56601		368	599	2.73E-34
GCF_001425265.1|WP_056397199.1609	CDD	cd07341	M56_BlaR1_MecR1_like	178	317	4.03119E-28
GCF_001425265.1|WP_056397199.1609	Pfam	PF05569	BlaR1 peptidase M56	20	316	5.3E-29

GCF_001425265.1|WP_056397199.1609	Gene3D	G3DSA:3.40.710.10		366	609	6.5E-81
GCF_001425265.1|WP_056397199.1609	Pfam	PF00905	Penicillin binding protein transpeptidase domain	378	600	2.4E-40
GCF_001758795.1|WP_071658926.1613	CDD	cd07341	M56_BlaR1_MecR1_like	123	292	3.41763E-31
GCF_001758795.1|WP_071658926.1613	Pfam	PF00905	Penicillin binding protein transpeptidase domain	385	600	3.0E-36
GCF_001758795.1|WP_071658926.1613	Pfam	PF05569	BlaR1 peptidase M56	12	294	2.0E-30
GCF_001758795.1|WP_071658926.1613	Gene3D	G3DSA:3.40.710.10		369	613	1.0E-78
GCF_001758795.1|WP_071658926.1613	SUPERFAMILY	SSF56601		372	598	1.11E-29
GCF_001425265.1|WP_056397197.1621	ProSitePatterns	PS00336	Beta-lactamase class-C active site.	304	311	-
GCF_001425265.1|WP_056397197.1621	Pfam	PF00905	Penicillin binding protein transpeptidase domain	25	227	4.6E-28
GCF_001425265.1|WP_056397197.1621	ProSitePatterns	PS00337	Beta-lactamase class-D active site.	48	58	-
GCF_001425265.1|WP_056397197.1621	Gene3D	G3DSA:3.40.710.10		22	251	2.9E-74
GCF_001425265.1|WP_056397197.1621	SUPERFAMILY	SSF56601		256	594	1.24E-84
GCF_001425265.1|WP_056397197.1621	SUPERFAMILY	SSF56601		17	230	5.5E-27
GCF_001425265.1|WP_056397197.1621	Pfam	PF00144	Beta-lactamase	256	593	5.9E-67
GCF_001425265.1|WP_056397197.1621	Gene3D	G3DSA:3.40.710.10		252	607	7.2E-118
GCF_001758785.1|WP_070245907.1632	ProSitePatterns	PS00337	Beta-lactamase class-D active site.	417	427	-
GCF_001758785.1|WP_070245907.1632	CDD	cd07341	M56_BlaR1_MecR1_like	124	300	1.9486E-31
GCF_001758785.1|WP_070245907.1632	Pfam	PF00905	Penicillin binding protein transpeptidase domain	407	623	3.5E-35
GCF_001758785.1|WP_070245907.1632	SUPERFAMILY	SSF56601		393	616	1.09E-30
GCF_001758785.1|WP_070245907.1632	Pfam	PF05569	BlaR1 peptidase M56	20	284	1.2E-27
GCF_001758785.1|WP_070245907.1632	Gene3D	G3DSA:3.40.710.10		390	631	1.4E-67
GCF_000335815.1|WP_008451277.1601	Gene3D	G3DSA:3.40.710.10		357	601	3.6E-78
GCF_000335815.1|WP_008451277.1601	CDD	cd07341	M56_BlaR1_MecR1_like	123	308	4.69834E-32
GCF_000335815.1|WP_008451277.1601	Pfam	PF00905	Penicillin binding protein transpeptidase domain	373	588	1.5E-34
GCF_000335815.1|WP_008451277.1601	Pfam	PF05569	BlaR1 peptidase M56	12	288	2.3E-31
GCF_000335815.1|WP_008451277.1601	SUPERFAMILY	SSF56601		359	586	6.91E-30
GCF_000381565.1|WP_026334185.1605	Pfam	PF00905	Penicillin binding protein transpeptidase domain	375	588	3.2E-37
GCF_000381565.1|WP_026334185.1605	Pfam	PF05569	BlaR1 peptidase M56	12	293	5.9E-29
GCF_000381565.1|WP_026334185.1605	SUPERFAMILY	SSF56601		367	585	4.1E-32
GCF_000381565.1|WP_026334185.1605	CDD	cd07341	M56_BlaR1_MecR1_like	123	262	9.10106E-28
GCF_000381565.1|WP_026334185.1605	Gene3D	G3DSA:3.40.710.10		362	605	2.6E-77

GCF_900143065.1|WP_072783046.1597	Pfam	PF05569	BlaR1 peptidase M56	18	271	2.0E-30
GCF_900143065.1|WP_072783046.1597	ProSitePatterns	PS00337	Beta-lactamase class-D active site.	382	392	-
GCF_900143065.1|WP_072783046.1597	Gene3D	G3DSA:3.40.710.10		355	597	2.2E-71
GCF_900143065.1|WP_072783046.1597	CDD	cd07341	M56_BlaR1_MecR1_like	134	305	6.64842E-27
GCF_900143065.1|WP_072783046.1597	Pfam	PF00905	Penicillin binding protein transpeptidase domain	345	590	5.6E-34
GCF_900143065.1|WP_072783046.1597	SUPERFAMILY	SSF56601		358	589	1.09E-28
GCF_000383895.1|WP_019923896.1631	CDD	cd07341	M56_BlaR1_MecR1_like	143	322	7.635E-28
GCF_000383895.1|WP_019923896.1631	Pfam	PF05569	BlaR1 peptidase M56	21	287	1.1E-27
GCF_000383895.1|WP_019923896.1631	Pfam	PF00905	Penicillin binding protein transpeptidase domain	405	622	2.2E-33
GCF_000383895.1|WP_019923896.1631	Gene3D	G3DSA:3.40.710.10		389	630	2.6E-68
GCF_000383895.1|WP_019923896.1631	SUPERFAMILY	SSF56601		392	615	3.7E-29
GCF_000383895.1|WP_019923896.1631	ProSitePatterns	PS00337	Beta-lactamase class-D active site.	416	426	-

EMBOSS pepwindowall

ClustalO alignment

GCF_001758545.1|WP_071849985.1, GCF_001425265.1|WP_056397197.1 and GCF_900129765.1|WP_073220251.1 are three fusion proteins between a class C and a class D beta-lactamase.

```
CLUSTAL O(1.2.4) multiple sequence alignment


GCF_900143065.1|WP_072783046.1      --------MTGFDIALVRLLLAAAGSLAAGGAVWGVALLCRRYLP---ALAQHRSLWLLG  49
GCF_001423125.1|WP_056159201.1      MIFGGIDTGIDLDLTLARLLLAAGGSLAVGCAVWAAALLCRRWLP---VLTQQRSLWLGA  57
GCF_000383895.1|WP_019923896.1      ------MMPIDLDIVLARLLLAAAGSLAAGGAVWAVAVLCRRTLP---ALAQQRSLWLSG  51
GCF_001758785.1|WP_070245907.1      ------MMPIDLDVVLARLLLAAAGSLAAGGAVWAVAVLCRRTLP---ALAQQRSLWLCG  51
GCF_001191005.1|WP_050407176.1      --------MSSFDLVLLRFLLASLGCLLAGLSVWGLSALLRRYLP---ALAAQRSIWLLG  49
GCF_000315425.1|WP_005669363.1      --------MNPLDQLVIRFLFAGAGCLAAGLAVWGATWLARRALP---ALGMQRSTWLLG  49
GCF_000381565.1|WP_026334185.1      --------MAFSTLFICRFLLASLGCMAAGLAVWALTAACRRHLP---QVAMQRSTWLLG  49
GCF_001758795.1|WP_071658926.1      --------MAISTLLLFRFLLASLGCMAAGLVVWALTAACRRCLP---QVAVQRSTWLLG  49
GCF_001758545.1|WP_071849986.1      --------MAISTLLIFRFLLASLGCMAAGLVVWALTAACRRYLP---QVALQRSTWLLG  49
GCF_000335815.1|WP_008451277.1      --------MAISTLLIFRFLLASLGCLAAGLVVWALTAACRRYLP---QVAVQRSTWLLG  49
GCF_000344615.1|WP_017879576.1      --------MSAFDLWLLRFLLASCGCVVAGLGVWALTALCRRCVA---EFSLQRSMWLLS  49
GCF_900129765.1|WP_073218605.1      --MTGL--SALLDQLPLRFLLASAACVAVGMGAWGVTAMFGRL-P---GVALRRSTWLLG  52
GCF_001425045.1|WP_057157847.1      --MTG---AVLADAWLLRFLLASAGCLAAGLAVWALTALCRRL-P---GVALHRSVWLLS  51
GCF_001425265.1|WP_056397199.1      --MSG---TAPLDAWLLRFLLASAGCLAAGLGVWALTALCRRL-P---GVALQRSTWLLG  51
GCF_001758545.1|WP_071849985.1      ------------------------------------------------------------  0
GCF_001425265.1|WP_056397197.1      ------------------------------------------------------------  0
GCF_900129765.1|WP_073220251.1      ------------------------------------------------------------  0
sp|P12287|BLAR_BACLI                --MS----SS----FFIPFLVSQIL----LSLFFSIIILIKKLLRTQITVGTHYYISVIS  46


GCF_900143065.1|WP_072783046.1      QIAVAVVFAAMLLPTTQRLRVVPVIEMNEEAPSPMPAASASPQASA----TVTPAADLTP  105
GCF_001423125.1|WP_056159201.1      QVAVAVVFLAMLLPPSDSLRVLPLIEIVEPEAAPASAVPAAAAATHA-AEATRPAPDLVA  116
GCF_000383895.1|WP_019923896.1      QVAVAAVFLAMLWPPAESLRVMPIIEIVEPAAAPMPATPASAEAHAAMAAAPAPGLGLTS  111
GCF_001758785.1|WP_070245907.1      QVAVAAVFLAMLLPPDEGLRVMPIIEIVEQEAAPAAPMPAVPPA----QAAAAPVLALAS  107
GCF_001191005.1|WP_050407176.1      QLTVIGTFVLILLPHSERVRLLLPIEGATETVSHYLVPAA-PAA------APS-QPVAPL  101
GCF_000315425.1|WP_005669363.1      QVAIAATFLLLLAPPAQQAPMHPVFEVDVGAAAA----SL-PAA------LAPPGLAAGV  98
GCF_000381565.1|WP_026334185.1      QLTIIATFLALLLPHSERLRLLPAIELPETMLAAPAAPDH-ATA------PAQDAAMAGG  102
GCF_001758795.1|WP_071658926.1      QMTIIATFLVILLPHSERLRLLPAIELPETMLAAAAAPDH-AAT------PAPAAAVADS  102
GCF_001758545.1|WP_071849986.1      QMTIIATFLVILLPHSERLRLLPAIELPETMLAAPAAPEH-AAT------PPHAAAASDD  102
GCF_000335815.1|WP_008451277.1      QMTIIATFLVILLPHSERLRLLPPIELPETVPAAPATPEH-AAT------PAPAAAVADS  102
GCF_000344615.1|WP_017879576.1      QITVVATFLVILLPHSERLRVIPPIDLADETAARLVAAGG-TTA------RAAASTAVSA  102
GCF_900129765.1|WP_073218605.1      QVTVMAAFLVVLLPHSEHLRLVPPIDLPEVALSHATATDG-GSA------LPGPGRPSAA  105
GCF_001425045.1|WP_057157847.1      QATVVAAFLVILLPHSERLRLVPPIELPEAAASRPAAQDG-PAA------RRAGEGSDAA  104
GCF_001425265.1|WP_056397199.1      QATVVAAFLVILLPHSERLRLVPPIDLPDAAQVRPAASGG-QAA------QPAAAGQDAL  104
GCF_001758545.1|WP_071849985.1      ------------------------------------------------------------  0
GCF_001425265.1|WP_056397197.1      ------------------------------------------------------------  0
GCF_900129765.1|WP_073220251.1      ------------------------------------------------------------  0
sp|P12287|BLAR_BACLI                LLALIAPFIPFHFLKSHHFDWILNLGGAQSALSQTHSTD-------------------KT  87
```

```
GCF_900143065.1|WP_072783046.1      AAPALSWHTWLRD------------AARAWLLLYLLGLGHAVWRWRRAQR----WLEALA  149
GCF_001423125.1|WP_056159201.1      VAADRPASAWMRD------------AGRAWLLLYLSGLAHALWRWWRAQR----LLDTLA  160
GCF_000383895.1|WP_019923896.1      SAADRPLSAWVRD------------AGRAWLVLYLLGLLHTLWRWQRAQR----LLEALA  155
GCF_001758785.1|WP_070245907.1      AATERPLSAWARD------------AGRAWLVLYLLGLVHALWRWRRAQR----LLDGLA  151
GCF_001191005.1|WP_050407176.1      VQAQREEHPWLNL------------AAYAWLAAYLLGLAYTVGRLLHGQR----MLNRLA  145
GCF_000315425.1|WP_005669363.1      PASIAGERSWLAW------------LAWAWASVYACGLAWTLGRLWRGQR----IVQRLL  142
GCF_000381565.1|WP_026334185.1      HSAAADHRAWLSH------------GAQAWLSLYLLGLAYTTGRVLQSQR----TLNGLA  146
GCF_001758795.1|WP_071658926.1      GDAPTDYRAWLTG------------GAQAWLSLYLLGLAYTTGRVLQAHR----TLNGLA  146
GCF_001758545.1|WP_071849986.1      GKAATDYRAWLTG------------GAQAWLSLYLLGLAYTTGRVLQAHR----TLNGLA  146
GCF_000335815.1|WP_008451277.1      GNAATDYRAWLTG------------GAQAWLSLYLLGLAYTTGRVLQAQR----TLNALA  146
GCF_000344615.1|WP_017879576.1      PVAGSIERPWLAY------------GAQAWLLVYLLGLGYAGARLLHARR----ILNGLC  146
GCF_900129765.1|WP_073218605.1      TGDALRPAIWLTR------------AAQVWLLVYLLGLGYTVLRLLYARR----LLDHLA  149
GCF_001425045.1|WP_057157847.1      STGQVQPASWLTR------------AAQAWLLAYLLGLGYTVFQLLRARR----MLNGLA  148
GCF_001425265.1|WP_056397199.1      AEAGTRPAAWLTH------------AAQAWLLAYLLGLGYAVFQLLRARR----ILNGLA  148
GCF_001758545.1|WP_071849985.1      ------------------------------------------------------------  0
GCF_001425265.1|WP_056397197.1      ------------------------------------------------------------  0
GCF_900129765.1|WP_073220251.1      ------------------------------------------------------------  0
sp|P12287|BLAR_BACLI                TEAIGQHVNWVQDFSLSIEQSSSKMIDSAFFAVWILGVAVMLLATLYSNLKIGKIKKNLQ  147

GCF_900143065.1|WP_072783046.1      ASGSPLDAAA-HVPL----------------PDVIEVAAPISPMLQGLRKPRLLLPRHLR  192
GCF_001423125.1|WP_056159201.1      ASGRALTGAD-HAGLAPD------Q--QALPLPVIEVEVPMSPMLLGLFRPRLLLPRHLR  211
GCF_000383895.1|WP_019923896.1      ASGRALGASE-HAGFAQH------TTAQVSPLAVVEVDVAMSPMLLGLFRPRLLLPRHLR  208
GCF_001758785.1|WP_070245907.1      ASGLPLAVSE-HKGFAQH------SRTQ-VPLAVVEVDVPMSPMLLGLFRPRLLLPRHLR  203
GCF_001191005.1|WP_050407176.1      GSGHGLPQDEAHAGFGSEL-------ARASRAQVIEVDAPISPMLLGPLRPRLLLPRHLR  198
GCF_000315425.1|WP_005669363.1      RCGAP--------------------QAHPAYPAVIEVDAPIPPMLVGPFKPRLLLPRGLR  182
GCF_000381565.1|WP_026334185.1      ATGERLVPPGRHQGLDAA--------PPPPSLAVIEVDAPISPMLFGWFRPRLMLPRHLR  198
GCF_001758795.1|WP_071658926.1      ATGERLILAGRHPGLDAAA-------TRPPSLTVIEVDAPISPMLFGWFRPRLLLPRHLR  199
GCF_001758545.1|WP_071849986.1      ATGERLATPGLHHGLDAAA-------TPAPSLAIIEVDAPISPMLFGWFRPRLLLPRHLR  199
GCF_000335815.1|WP_008451277.1      ATGERLILAGRHPGLDAAA-------TPPPALAIIEVDAPISPMLFGWFRPRLLLPRHLR  199
GCF_000344615.1|WP_017879576.1      AAGCRVDALNQHDGFA---------ATLARAPAVIEVDAPISPMLFGLFRPRLLLPRHLR  197
GCF_900129765.1|WP_073218605.1      ASGVRLPAQPGTE------------TAPTSLPSIIEVDAPISPMLFGLLNPRLLLPRHLR  197
GCF_001425045.1|WP_057157847.1      ASGSRLPAPIPAASQ----------PAPAAAPIVIEVDAPISPMLFGLRKPRLLLPRHLR  198
GCF_001425265.1|WP_056397199.1      ASGQHLPAPPSASASAAAAAAAPAAGRPVRTPAVIEVDAPISPMLFGLFQPRLLLPRHLR  208
GCF_001758545.1|WP_071849985.1      ------------------------------------------------------------  0
GCF_001425265.1|WP_056397197.1      ------------------------------------------------------------  0
GCF_900129765.1|WP_073220251.1      ------------------------------------------------------------  0
sp|P12287|BLAR_BACLI                IVNNKELLSLFHTCKE--------EIRFHQKVILSRSPLIKSPITFGVIRPYIILPKDIS  199
```

```
GCF_900143065.1|WP_072783046.1      TFDPLQQQLIVEHELTHWRRHDLCWSVAAFALQSLFWFNPFMRLLRARLGWAQEFGCDRD  252
GCF_001423125.1|WP_056159201.1      EVEVFQQRLIVAHELTHWRRRDLHWSAAALVLQSLFWFNPFMRLLGARLGWAQEFGCDRD  271
GCF_000383895.1|WP_019923896.1      GFDTLQQQLIVEHELTHWRRRDLHWSAAALLLQSLFWFNPFMRLLGARLGWAQEFGCDRD  268
GCF_001758785.1|WP_070245907.1      GFDTLQQQLIVEHELTHWRRRDLHWSAAALLLQSLFWFNPFMRLLGARLGWAQEFGCDRD  263
GCF_001191005.1|WP_050407176.1      EFDAMQQQMIVEHELTHLRRRDLQWMTLGLVLQTLLWFNPFMRLLRASLGWAQELGCDRD  258
GCF_000315425.1|WP_005669363.1      DIDPLQRELIVAHELTHWRRGDLWWLTVGAALQALCWFNPAMRLLRDKLAWAQELGCDRD  242
GCF_000381565.1|WP_026334185.1      SFEPLQQQMIVEHELMHLRRHDLQWMSAGIVLQTLLWFNPFMRLLRDKLAWAQELGCDRD  258
GCF_001758795.1|WP_071658926.1      SFDPGQQQMIVEHELTHLRRHDLQWMSAGIVLQTLLWFNPFMRLLRDNLAWAQELGCDRD  259
GCF_001758545.1|WP_071849986.1      SFDPAQQQMIVEHELTHLRRHDLQWMSAGIVLQTLLWFNPFMRLLRDNLAWAQELGCDRD  259
GCF_000335815.1|WP_008451277.1      SFDPEQQQMIVEHELTHLRRHDLQWMSAGIVLQTLLWFNPFMRLLRDNLAWAQELGCDRD  259
GCF_000344615.1|WP_017879576.1      GFDVLQQQMIVEHELTHLRRRDLHWMSAGVLLQTLLWFNPFMRLLRAKLSWAQELGCDRD  257
GCF_900129765.1|WP_073218605.1      GFDAKQQQLIIEHELMHWRRRDLHWMSVGIALQSLLWFNPFMRMLRNRLSWAQELGCDRD  257
GCF_001425045.1|WP_057157847.1      SFDAAQQQLIVEHELTHWRRRDLQWMSIGIVLQTLLWFNPFMRLLRSSLSWAQELGCDRD  258
GCF_001425265.1|WP_056397199.1      SFDPAQQQLIVEHELTHWRRRDLQWMSVGIALQTLLWFNPFMRLLRGSLSWAQELGCDRD  268
GCF_001758545.1|WP_071849985.1      ------------------------------------------------------------  0
GCF_001425265.1|WP_056397197.1      ------------------------------------------------------------  0
GCF_900129765.1|WP_073220251.1      ------------------------------------------------------------  0
sp|P12287|BLAR_BACLI                MFSADEMKCVLLHELYHCKRKDMLINYFLCLLKIVYWFNPLVWYLSKEAKTEMEISCDFA  259

GCF_900143065.1|WP_072783046.1      VLRGRPPAERKAYAAALVAQFKLQLRP------------ADMALAFGASDAGA---HAPT  297
GCF_001423125.1|WP_056159201.1      VLRGRPPAERKAYAAALVAQLRWQHRP------------AGMALAFGAHDGGA---GTST  316
GCF_000383895.1|WP_019923896.1      VLRGRPPAERKAYAAALVAQLKLQYRP------------AGMALAFGASEANG--GHAPT  314
GCF_001758785.1|WP_070245907.1      VLRGRPSAERKAYAAALVAQLKLQCRP------------AGMALAFGASDAGSARTDAPT  311
GCF_001191005.1|WP_050407176.1      VLRGRPAAQRKVYAAALLAQLKLQVRP------------PEMALAFGSI-------DAST  299
GCF_000315425.1|WP_005669363.1      VLRGRPSFERRAYAAALLAQLRMQHRV------------VHGALAFGGV-------SPDT  283
GCF_000381565.1|WP_026334185.1      VLRHRPPALRRAYAAALVGQLRLQPHPATHAAT----HSATTALAFGGV-------SART  307
GCF_001758795.1|WP_071658926.1      VLRHRPSAQRKAYAAALVAQLRLQPHPATHPATHPATHLANTALAFGGV-------CART  312
GCF_001758545.1|WP_071849986.1      VLRNRPSAQRKAYAAALVAQLRLQPHPATHP--------ANTALAFGGV-------CART  304
GCF_000335815.1|WP_008451277.1      VLRNRPQAQRKAYAAALVAQLRLQPQSV------------KAALAFGGV-------SART  300
GCF_000344615.1|WP_017879576.1      VLRGRPQAQRKAYAAALVAQLRMQRGP------------TPAALAFGGV-------GAST  298
GCF_900129765.1|WP_073218605.1      VLRSRAPAQRKAYAAALVAQLRLQRDA------------RQTALAFGAV-------CAGT  298
GCF_001425045.1|WP_057157847.1      VLRGRPPAQRKAYAAALVAQLRLQHGT------------PKTALAFGGV-------CAST  299
GCF_001425265.1|WP_056397199.1      VLRGRPPAQRKAYAAALVAQLRLQHGA------------PKTALAFGGV-------CAST  309
GCF_001758545.1|WP_071849985.1      ------------------------------------------------------------  0
GCF_001425265.1|WP_056397197.1      ------------------------------------------------------------  0
GCF_900129765.1|WP_073220251.1      ------------------------------------------------------------  0
sp|P12287|BLAR_BACLI                VLKTLDKKLHLKYGEVILKFTSIKQRTSSLL----------AASEFSSS--------YKH  301
```

```
GCF_900143065.1|WP_072783046.1      LAARISLIRTPTTERRR---WPRWLALASLTAVAVVSVALQPALGWRSAEVQ--------  346
GCF_001423125.1|WP_056159201.1      LAARIGLIRTPATARGA---WPRALALASLAAVAVANFALQPALAWQAAGPAIEPRPLLA  373
GCF_000383895.1|WP_019923896.1      LAARIGLIRTPATARGA---WSRWVALGSLAAVAIANFALQPALAWQAAEPAIEPVRLLA  371
GCF_001758785.1|WP_070245907.1      LAARIGLIRTPATAR-A---WPRWVALASLAAVALANFALQPALAWQAAEPVIEPARLLA  367
GCF_001191005.1|WP_050407176.1      LASRLALIRQPGSALRGR--WARWAGVAALAGLAAGNFALQSALAGNVAPDLE-------  350
GCF_000315425.1|WP_005669363.1      LAARVELIRKPGAARHAA--WARGAGLAILALAFGGNLALQPALAWSNPPA---------  332
GCF_000381565.1|WP_026334185.1      LATRISLIREPAAAPRGP--WARGAAVAGLAGVFAASLAFQPALADRA--PVQ-------  356
GCF_001758795.1|WP_071658926.1      VAARISLIREPGATPRGP--WARAATITGLAGVFATSLAFQPALADRAATPAH-------  363
GCF_001758545.1|WP_071849986.1      VAARISLIREPGATPRGP--WARAATITGLAGVFATSLAFQPALADRSA-PAL-------  354
GCF_000335815.1|WP_008451277.1      VAARISLIREPGATPRGA--WARAATITGLAGVFATSLAFQPALADRAATPAL-------  351
GCF_000344615.1|WP_017879576.1      LAQRIALIRQPGTASRRP--WGRCAALAGLAGIVGATLAFQPALAWRIDPVAAAGPALDK  356
GCF_900129765.1|WP_073218605.1      LAARIALIREPSNGRSRGADAARIFSIAALASLFGASLAFQPALAWRSDPSVA-------  351
GCF_001425045.1|WP_057157847.1      LASRIALIREPGRAQGRRARAARLLALAGLACVFAASLAFQPALAWRIAPAAS-------  352
GCF_001425265.1|WP_056397199.1      LASRIALIREPARAQGRRARAARLLALAGLAGVFAASLALQPALAWRIAPAAS-------  362
GCF_001758545.1|WP_071849985.1      -------------------MNFRHIILG--------------------------------  9
GCF_001425265.1|WP_056397197.1      --------------------MIAMAALG--------------------------------  8
GCF_900129765.1|WP_073220251.1      ------------------------------------------------------------  0
sp|P12287|BLAR_BACLI                IKRRIVTVVNFQTAS--PLLKAK--SALVFTLVLGAILAGTPSVSILA---MQKETRFLP  354


GCF_900143065.1|WP_072783046.1      --------------DASAGAALALDCTVMVDAANGASLVREGTCGERVTPASTFKIAISL  392
GCF_001423125.1|WP_056159201.1      SPAVND-----ARLAAAPAAPAVIDCTVMVDAASGATLVREGTCDARVTPASTFKIAISL  428
GCF_000383895.1|WP_019923896.1      RHSP-----DIAAAQPIATASATLDCTMMVDAASGAALVREGTCDASVTPASTFKIAISL  426
GCF_001758785.1|WP_070245907.1      GHNPAQHSPDTTAPPAAATTPASLDCTMLVDAASGVALVREGTCDASVTPASTFKIAISL  427
GCF_001191005.1|WP_050407176.1      -------------------ALAAIRCTQLMDAASGRVLQREGQCEARVTPASTFNIAVSL  391
GCF_000315425.1|WP_005669363.1      ----------------------SPDCTLMLDAASGARLVEEGDCDVRATPASTFNIAVSL  370
GCF_000381565.1|WP_026334185.1      -----------------AAAPATFSCTDMVDAASGAQLLRDGHCDERVTPASTFNIAVSL  399
GCF_001758795.1|WP_071658926.1      -----------------AATPATFSCTEMVDAASGKRLVHDGLCDERVTPASTFNIAVSL  406
GCF_001758545.1|WP_071849986.1      -----------------AATPATFSCTEMVDAASGKRLVHDGLCDERVTPASTFNIAVSL  397
GCF_000335815.1|WP_008451277.1      -----------------AATPATFSCTEMVDAASGKRLVHDGLCDERVTPASTFNIAVSL  394
GCF_000344615.1|WP_017879576.1      ----------ALWPFTPATPQGTISCTELVDAASGERLVHEGQCEQRVTPASTFNIPVSL  406
GCF_900129765.1|WP_073218605.1      --------------------QAAFSCTLLVDAASGAQLVRDGHCDEQVTPASTFNIPVAL  391
GCF_001425045.1|WP_057157847.1      --------------------QVPFTCTVIADAASGRQLAREGHCDERVTPASTFNIPVAL  392
GCF_001425265.1|WP_056397199.1      --------------------QAPFSCTVLADAASGQAPAREGHCDERVTPASTFNIVVAL  402
GCF_001758545.1|WP_071849985.1      ---------ALASLVPISAAHATEVCTALADS-NGPTLFQRGDCQRQVTAASTFKIAISL  59
GCF_001425265.1|WP_056397197.1      ---------CVAGLAG-VPAHGAEICTAIADAATGKVLMQRGDCQRQVTPASTFKIPLSL  58
GCF_900129765.1|WP_073220251.1      --------------------------------------MQRGDCQRQVTPASTFKIPLSL  22
sp|P12287|BLAR_BACLI                GTNVEYE--DYSTFFDKFSASG--GFVLFNSNRKKYTIYNRKESTSRFAPASTYKVFSAL  410
                                                                           .   .    : ***:::  :*
```

```
GCF_900143065.1|WP_072783046.1      MGFDSGVLRDEHAPYLPYQESYASSNPSWRHGTDPAGWLRESIVWYSQQVTSQLGAGSVR  452
GCF_001423125.1|WP_056159201.1      MGFDSGVLRDDHAPYLPYKASYASSNPSWRHGTDPAGWLRESIVWYSQQVTRRLGAASVR  488
GCF_000383895.1|WP_019923896.1      MGFDSGVLRDDHAPYLPYKASYASPNPSWRHGTDPAGWLRESIVWYSQQVTKRLGPASVR  486
GCF_001758785.1|WP_070245907.1      MGFDSGVLRDDHAPYLPYKASYASSNPGWRHGTDPAGWLRESIVWYSQQVTKRLGAASVR  487
GCF_001191005.1|WP_050407176.1      MGYDSGFLRDEHTPVLPFKEGYPAWIPEWRQDLDPSGWIKYSSVWYAQQVTRQLGAARFQ  451
GCF_000315425.1|WP_005669363.1      LGYDAGILVDAHTPALPFKPGYIDWLPAWRATTDPTSWIRSSTVWYAQQVTARLGLDGLQ  430
GCF_000381565.1|WP_026334185.1      MGYDSGILRDAHSPSLPFKPGYADWNPDWRATTDPASWIRNSTVWYAQQVTASLGAQRFR  459
GCF_001758795.1|WP_071658926.1      MGYDSGILRDAHAPSLPFKPGYIDWNPDWRATTDPTSWIRNSTVWYAQQVTAGLGARRFQ  466
GCF_001758545.1|WP_071849986.1      MGYDSGILRDAHSPSLPFKPGYIDWNPDWRATTDPTSWIRNSTVWYAQQVTAGLGARRFQ  457
GCF_000335815.1|WP_008451277.1      MGYDSGILRDAHSPSLPFKPGYIDWNPDWRATTDPTSWIRNSTVWYAQQVTAGLGARRFQ  454
GCF_000344615.1|WP_017879576.1      MGYDSGILRDEHTPKLPYRAGYVNWNPSWRAATDPTSWLKNSVLWYAQQVTLQLGAARFQ  466
GCF_900129765.1|WP_073218605.1      MGFDSGILQDEHAPMLPFKTGYPAYIPSWQADTDPSTWLQNSVLWYAQQITTRLGAKRFQ  451
GCF_001425045.1|WP_057157847.1      MGYDSGILQHEHAPLMPFKTGYPAYVPSWRADTDPSGWLHNSVLWYAQQVTATLGAARFQ  452
GCF_001425265.1|WP_056397199.1      MGYDSGILRDQHAPVLPFKAGYPAYIPSWRAATDPAGWLQNSVLWYAQQVTRQLGAARFQ  462
GCF_001758545.1|WP_071849985.1      MGYDAGILKDQRTPKLPFREGYVDWRADWRQDTDPTMWMTNSVVWYSQQVTQQLGMQRFA  119
GCF_001425265.1|WP_056397197.1      MGYDAGFLTDEHAPQLPFRRGDPDWRPSWRSATDPAKWMSESVVWYSQRITVALGQARFA  118
GCF_900129765.1|WP_073220251.1      MGYDAGFLKDTQTPELPFRQGYVDWRPSWRSATAPAKWMSESVVWYSQQITQSLGKKRFA  82
sp|P12287|BLAR_BACLI                LALESGIITKNDSHMTWDGTQY--PYKEWNQDQDLFSAMSSSTTWYFQKLDRQIGEDHLR  468
                                    :. ::*.: .  :               *.        :  *  ** *::   :*   .

GCF_900143065.1|WP_072783046.1      NYVQSFEYGNRDIASVAGVDDAVAFSELSPTLRISALEQAAFLRKVVNRSLPLSAHAYDM  512
GCF_001423125.1|WP_056159201.1      GYVQAFDYGNRDLSSVAGVTEAVAVSELSPTLRISPQEQTVFLRKVVNRKLPLSPHAYEA  548
GCF_000383895.1|WP_019923896.1      GYVQAFDYGNRNLASVAGVDDAVAVSELSPTLRITPQQQTDFLRKVVNRELAVSQQAYDV  546
GCF_001758785.1|WP_070245907.1      NYVRAFDYGNRTLASVAGVADAVAVSELSPTLRITPQQQTEFLRKVVNRELALSPQAYDV  547
GCF_001191005.1|WP_050407176.1      RYIADFGYGNRDVAGDAGADNGLGYAWINSSLKISGDEQVAFLGRMARRELPLQPQAYEM  511
GCF_000315425.1|WP_005669363.1      SYVRRFDYGNQDLSG------GVADAWIGSSLQISAQEQAAFLRKVVNRELGLNPHAYDM  484
GCF_000381565.1|WP_026334185.1      QYVRGFGYGNLDVSGDPGKDNGLAMSWIASSLKISPAEQTAFLRKIVNRQLPLSAHAYDM  519
GCF_001758795.1|WP_071658926.1      HYVNSFGYGNRDVSGDAGKDNGLAMAWIESSLKISATEQTAFLRKIVNRQLPLSAHAYDM  526
GCF_001758545.1|WP_071849986.1      QYLNSFGYGNLDVSGDAGKDNGLAMSWIASSLKISAAEQTAFLRKVVNRQLPLSAHAYDM  517
GCF_000335815.1|WP_008451277.1      QYLNSFDYGNLDVSGDAGKDNGLAMSWIASSLKISAAEQTAFLRKVVNRQLPLSAHAYDM  514
GCF_000344615.1|WP_017879576.1      RYVKDFHYGNHDVAGDAGKDNGLTLSWVSSSLKISPVEQVAFLRNVVNRELPLTAKAYDM  526
GCF_900129765.1|WP_073218605.1      DYVQGFSYGNQDLGGDPGKDNGLVQSWVSSSLRISPSEQVNFLRKVANRELPLSPQAYAK  511
GCF_001425045.1|WP_057157847.1      HYVQRFGYGNQDLSGDPGKDNGLSLAWVGSSLRISPLEQVAFLRKVANRELPLSAHAYAM  512
GCF_001425265.1|WP_056397199.1      QYVQRFGYGNQDLAGEPGQDNGLTQSWVGSSLRISPLEQVAFLRKVARRELPLSAHAYAM  522
GCF_001758545.1|WP_071849985.1      AYTSQFKYGNANVAGDAEHD-GLTLSWISSSLKISPLEQLDFLNKVVNRQLGVSAHAYDM  178
GCF_001425265.1|WP_056397197.1      AYTRRFEYGNADVAGDARND-GLTASWLGSSLRISPLGQLSFLGRVVNRQLGVSEKAYEM  177
GCF_900129765.1|WP_073220251.1      EYTTRFNYGNADVSGDAGHE-GLTAAWLESSLRISPLEQLSFLGKVVNRQLGVSEHAYAM  141
sp|P12287|BLAR_BACLI                HYLKSIHYGNEDFSVP-------ADYWLDGSLQISPLEQVNILKKFYDNEFDFKQSNIET  521
                                     *   : ***  ..             :  :*:*:   *  :* ..  ..: .
```

```
GCF_900143065.1|WP_072783046.1      TARLLKLDQPVNGWEVYGKTGTAAVRLPDGSEDQAQDIGWFVGWAVKDGRTVVFARLLQH  572
GCF_001423125.1|WP_056159201.1      TARLLKLDAMPAGWEVHGKTGTAPVQLADGRTDRDNNIGWFVGWTIRDGRTLVFARLMQY  608
GCF_000383895.1|WP_019923896.1      TARLLKVDAAPNGWEVHGKTGTAPVRLANGRADRDNNIGWFVGWTVRDGRKLVFARLMQH  606
GCF_001758785.1|WP_070245907.1      TARLLKVEETPNGWEVHGKTGTAPVRLADGSADKDNYIGWFVGWTIKDGRKLVFARLMQY  607
GCF_001191005.1|WP_050407176.1      SARLFKLASFANGWEVYGKTGTGYPVKADGKEDKTRAYGWFVGWAAKGGRTIVFAYLVQD  571
GCF_000315425.1|WP_005669363.1      TETLLRLPALPNGWDVYAKTGTAVLEQPKGAQDPPRSYGWFVGWARRDGRTIVFARLILD  544
GCF_000381565.1|WP_026334185.1      TARLTALGALPNGWQIHGKTGTASPVLADGGDDRRHSYGWFVGWASKGGRTVVFSRLVLE  579
GCF_001758795.1|WP_071658926.1      TARLTALGTLPNGWQLHGKTGTASPVLADGSDDPRHSYGWFVGWAAKDGRTVVFSRLVLA  586
GCF_001758545.1|WP_071849986.1      TARLTALGTLPNGWQIHGKTGTASPVLADGSDDPRHSYGWFVGWATKDGRTVVFSRLVLA  577
GCF_000335815.1|WP_008451277.1      TARLTALGALPNGWQLHGKTGTASPVLADGSDDPQHSYGWFVGWATKDGRTVVFSRLVLA  574
GCF_000344615.1|WP_017879576.1      TLRIMQSDTLANGWEVHGKTGTASPVLPDGRDDEAHQYGWFVGWAKKDGRTIVFARLAQD  586
GCF_900129765.1|WP_073218605.1      TERILPQQTLGNGWHVVGKTGTASALLPDGGDDATRQYGWYVGWAKKGRRTVVFARLVLD  571
GCF_001425045.1|WP_057157847.1      TARIMPQQTLANGWQVTGKTGTASALLPDGSEDGTRQYGWYVGWATKGQRTVVFARLAMD  572
GCF_001425265.1|WP_056397199.1      TESIMPRQTLANGWEVHGKTGTASALLPDGSEDGTRQYGWYVGWASKGQRTVVFARLVLD  582
GCF_001758545.1|WP_071849985.1      TARLTQRDQPLAGWRIHGKTGAAS------------GYGWYVGWATKGKRSFSFAHLMQR  226
GCF_001425265.1|WP_056397197.1      TARLTRYGQPVEGWSVNGKTGSGS------------GFGWYVGWAEKGGRKYVFARLIEK  225
GCF_900129765.1|WP_073220251.1      TAQLTQWGQSPDGWRIHGKTGSGD------------GYGWYVGWASKGARAYVFARLIQK  189
sp|P12287|BLAR_BACLI                VKDSIRL-EESNGRVLSGKTGTSVI-------NGELHAGWFIGYVETADNTFFFAVHIQG  573
                                                *  : .***:.               **::*:.    .   *:

GCF_900143065.1|WP_072783046.1      PVE--ADLYAGRQTRDAFLRELAQRVL---------------------------------  597
GCF_001423125.1|WP_056159201.1      PVQ--SAGYAGPKTRAAFLDELAQRSL---------------------------------  633
GCF_000383895.1|WP_019923896.1      PVT--SESYAGLKTREAFLGELAQRSL---------------------------------  631
GCF_001758785.1|WP_070245907.1      PAD--SNSYAGLKTRQAFLGELAQRTL---------------------------------  632
GCF_001191005.1|WP_050407176.1      QKE--EEGAAGPRLRAAVLNQLPAQLETL-------------------------------  598
GCF_000315425.1|WP_005669363.1      RQH--PDRAAGPRLKEAFLRELPSRLDAL-------------------------------  571
GCF_000381565.1|WP_026334185.1      DTQ--A-DAAGPRTRDAFLRELPAQLDTL-------------------------------  605
GCF_001758795.1|WP_071658926.1      DKQ--AGSAAGPRTRDAFLRDLPAQLDAL-------------------------------  613
GCF_001758545.1|WP_071849986.1      DKQ--AGSAAGPRTKDAFLHDLPAQLDAL-------------------------------  604
GCF_000335815.1|WP_008451277.1      DKQ--AGSAAGPRTRDAFLRDLPALLDAL-------------------------------  601
GCF_000344615.1|WP_017879576.1      PQR--QTGAAGPRAKAAFLRDLPARLDAL-------------------------------  613
GCF_900129765.1|WP_073218605.1      AKQ--ADAMGGARARAAMLRDLPPQLDRL-------------------------------  598
GCF_001425045.1|WP_057157847.1      AKQ--EGAMGGPRSREALLRELPARLDAF-------------------------------  599
GCF_001425265.1|WP_056397199.1      ARQ--EVAMGGARAREALLRELPARLDAL-------------------------------  609
GCF_001758545.1|WP_071849985.1      DDTQPKEVSTGVLAREALLKELPLLLGSVEQEALLRETVDQTILPLMKKYDVPGMALALT  286
GCF_001425265.1|WP_056397197.1      EQGEPQDVPAGVLARDGLVAEFPALANAIE--------VDQAFKPLLEKHGLPGMAVALS  277
GCF_900129765.1|WP_073220251.1      DKSDAADVPGGMLARDSLMEEFPALVNGIA--------VDQTMRPLMQENDIPGMAVAVS  241
sp|P12287|BLAR_BACLI                EKRAAG-----SSAAEIALSIL-------DKKG---------IYPSVSR-----------  601
                                                      :  :
```

```
GCF_900143065.1|WP_072783046.1      ------------------------------------------------------------ 597
GCF_001423125.1|WP_056159201.1      ------------------------------------------------------------ 633
GCF_000383895.1|WP_019923896.1      ------------------------------------------------------------ 631
GCF_001758785.1|WP_070245907.1      ------------------------------------------------------------ 632
GCF_001191005.1|WP_050407176.1      ------------------------------------------------------------ 598
GCF_000315425.1|WP_005669363.1      ------------------------------------------------------------ 571
GCF_000381565.1|WP_026334185.1      ------------------------------------------------------------ 605
GCF_001758795.1|WP_071658926.1      ------------------------------------------------------------ 613
GCF_001758545.1|WP_071849986.1      ------------------------------------------------------------ 604
GCF_000335815.1|WP_008451277.1      ------------------------------------------------------------ 601
GCF_000344615.1|WP_017879576.1      ------------------------------------------------------------ 613
GCF_900129765.1|WP_073218605.1      ------------------------------------------------------------ 598
GCF_001425045.1|WP_057157847.1      ------------------------------------------------------------ 599
GCF_001425265.1|WP_056397199.1      ------------------------------------------------------------ 609
GCF_001758545.1|WP_071849985.1      DHGKNYVFNYGLASRETRQPVDRDTLFEVGSVSKTLVATLATYAQAQGRLALSDKVSQHM 346
GCF_001425265.1|WP_056397197.1      VNGKHYFYNYGVASQETGQPVSEATLFELGSVSKTFTVTLAAYAQAQGRLALTDPVSRHL 337
GCF_900129765.1|WP_073220251.1      VNGKHYFYHYGVASKETGQPVTNATLFEIGSLSKTFTATLATYAQAQGKLAMTDAVSQHV 301
sp|P12287|BLAR_BACLI                ------------------------------------------------------------ 601

GCF_900143065.1|WP_072783046.1      ------------------------------------------------------------ 597
GCF_001423125.1|WP_056159201.1      ------------------------------------------------------------ 633
GCF_000383895.1|WP_019923896.1      ------------------------------------------------------------ 631
GCF_001758785.1|WP_070245907.1      ------------------------------------------------------------ 632
GCF_001191005.1|WP_050407176.1      ------------------------------------------------------------ 598
GCF_000315425.1|WP_005669363.1      ------------------------------------------------------------ 571
GCF_000381565.1|WP_026334185.1      ------------------------------------------------------------ 605
GCF_001758795.1|WP_071658926.1      ------------------------------------------------------------ 613
GCF_001758545.1|WP_071849986.1      ------------------------------------------------------------ 604
GCF_000335815.1|WP_008451277.1      ------------------------------------------------------------ 601
GCF_000344615.1|WP_017879576.1      ------------------------------------------------------------ 613
GCF_900129765.1|WP_073218605.1      ------------------------------------------------------------ 598
GCF_001425045.1|WP_057157847.1      ------------------------------------------------------------ 599
GCF_001425265.1|WP_056397199.1      ------------------------------------------------------------ 609
GCF_001758545.1|WP_071849985.1      PALRGSSFDHIKLIHLGTHTAGEFPMQVPGNIKNYDQLMDYYRSWQQPASAAGASRTYSN 406
GCF_001425265.1|WP_056397197.1      PALRGSVFDRVSLVHLGTHTAGDFPLQLPQEITTHAQLMAYYKGWQ-PGHAPGSHRTYSN 396
GCF_900129765.1|WP_073220251.1      PQLRGSNFDHIQLLHLGTHTVGDFPMQVPLDIKTYDQLMDYYKRWQ-PGHGAGTHRTYSN 360
sp|P12287|BLAR_BACLI                ------------------------------------------------------------ 601
```

```
GCF_900143065.1|WP_072783046.1      ------------------------------------------------------------ 597
GCF_001423125.1|WP_056159201.1      ------------------------------------------------------------ 633
GCF_000383895.1|WP_019923896.1      ------------------------------------------------------------ 631
GCF_001758785.1|WP_070245907.1      ------------------------------------------------------------ 632
GCF_001191005.1|WP_050407176.1      ------------------------------------------------------------ 598
GCF_000315425.1|WP_005669363.1      ------------------------------------------------------------ 571
GCF_000381565.1|WP_026334185.1      ------------------------------------------------------------ 605
GCF_001758795.1|WP_071658926.1      ------------------------------------------------------------ 613
GCF_001758545.1|WP_071849986.1      ------------------------------------------------------------ 604
GCF_000335815.1|WP_008451277.1      ------------------------------------------------------------ 601
GCF_000344615.1|WP_017879576.1      ------------------------------------------------------------ 613
GCF_900129765.1|WP_073218605.1      ------------------------------------------------------------ 598
GCF_001425045.1|WP_057157847.1      ------------------------------------------------------------ 599
GCF_001425265.1|WP_056397199.1      ------------------------------------------------------------ 609
GCF_001758545.1|WP_071849985.1      LTIGLLGMISAQSMGLPIADAMEKQLLPALGMRQTYIKVPADQMTHYAQGYNDANAPVRV 466
GCF_001425265.1|WP_056397197.1      PGIGLLSLATAASLGVPYADAVEQTLFPALGLAHSYLRVPAGQMAQYAQGYNSKGAPVRM 456
GCF_900129765.1|WP_073220251.1      LGIGLLSIATAHSLGMPYVDAVEQTLLPALGLKHTWIKVPADEMAQYAQGYNSKGAPVRV 420
sp|P12287|BLAR_BACLI                ------------------------------------------------------------ 601

GCF_900143065.1|WP_072783046.1      ------------------------------------------------------------ 597
GCF_001423125.1|WP_056159201.1      ------------------------------------------------------------ 633
GCF_000383895.1|WP_019923896.1      ------------------------------------------------------------ 631
GCF_001758785.1|WP_070245907.1      ------------------------------------------------------------ 632
GCF_001191005.1|WP_050407176.1      ------------------------------------------------------------ 598
GCF_000315425.1|WP_005669363.1      ------------------------------------------------------------ 571
GCF_000381565.1|WP_026334185.1      ------------------------------------------------------------ 605
GCF_001758795.1|WP_071658926.1      ------------------------------------------------------------ 613
GCF_001758545.1|WP_071849986.1      ------------------------------------------------------------ 604
GCF_000335815.1|WP_008451277.1      ------------------------------------------------------------ 601
GCF_000344615.1|WP_017879576.1      ------------------------------------------------------------ 613
GCF_900129765.1|WP_073218605.1      ------------------------------------------------------------ 598
GCF_001425045.1|WP_057157847.1      ------------------------------------------------------------ 599
GCF_001425265.1|WP_056397199.1      ------------------------------------------------------------ 609
GCF_001758545.1|WP_071849985.1      HPAVLEPEAYGIKTTAADLIRFVDANLGQAALDEALRQAVEATHIGYFKVGKMTQDLIWE 526
GCF_001425265.1|WP_056397197.1      NPGVLAEEAYGVKSTTRDLIRFVDANMGLLPLEDKLARAVAATHTGYFKTGAMTQDLVWE 516
GCF_900129765.1|WP_073220251.1      NPGVLADEAYGVKSTAADLIHFLDANMGLITLDANLARAIRDTHAGYFKAGPMTQDLVWE 480
sp|P12287|BLAR_BACLI                ------------------------------------------------------------ 601
```

```
GCF_900143065.1|WP_072783046.1      ------------------------------------------------------------ 597
GCF_001423125.1|WP_056159201.1      ------------------------------------------------------------ 633
GCF_000383895.1|WP_019923896.1      ------------------------------------------------------------ 631
GCF_001758785.1|WP_070245907.1      ------------------------------------------------------------ 632
GCF_001191005.1|WP_050407176.1      ------------------------------------------------------------ 598
GCF_000315425.1|WP_005669363.1      ------------------------------------------------------------ 571
GCF_000381565.1|WP_026334185.1      ------------------------------------------------------------ 605
GCF_001758795.1|WP_071658926.1      ------------------------------------------------------------ 613
GCF_001758545.1|WP_071849986.1      ------------------------------------------------------------ 604
GCF_000335815.1|WP_008451277.1      ------------------------------------------------------------ 601
GCF_000344615.1|WP_017879576.1      ------------------------------------------------------------ 613
GCF_900129765.1|WP_073218605.1      ------------------------------------------------------------ 598
GCF_001425045.1|WP_057157847.1      ------------------------------------------------------------ 599
GCF_001425265.1|WP_056397199.1      ------------------------------------------------------------ 609
GCF_001758545.1|WP_071849985.1      QYPAAAGLPGLLVSASEQVTWKSNPATPLTPPLAPQADALLHKTGSTGGFGAYVLFSPGR 586
GCF_001425265.1|WP_056397197.1      QYPGHAGLDQLLVSTAEKVVFEPNPATEITPPLPPQADAWLHKTGSTGGFSAYVLFNPAR 576
GCF_900129765.1|WP_073220251.1      QYPSQAALEQLLVSTSEKMTRESNPVSTIAPPLPPQAHAWLHKTGSTGGFSAYALFNPAR 540
sp|P12287|BLAR_BACLI                ------------------------------------------------------------ 601

GCF_900143065.1|WP_072783046.1      --------------------------------------------- 597
GCF_001423125.1|WP_056159201.1      --------------------------------------------- 633
GCF_000383895.1|WP_019923896.1      --------------------------------------------- 631
GCF_001758785.1|WP_070245907.1      --------------------------------------------- 632
GCF_001191005.1|WP_050407176.1      --------------------------------------------- 598
GCF_000315425.1|WP_005669363.1      --------------------------------------------- 571
GCF_000381565.1|WP_026334185.1      --------------------------------------------- 605
GCF_001758795.1|WP_071658926.1      --------------------------------------------- 613
GCF_001758545.1|WP_071849986.1      --------------------------------------------- 604
GCF_000335815.1|WP_008451277.1      --------------------------------------------- 601
GCF_000344615.1|WP_017879576.1      --------------------------------------------- 613
GCF_900129765.1|WP_073218605.1      --------------------------------------------- 598
GCF_001425045.1|WP_057157847.1      --------------------------------------------- 599
GCF_001425265.1|WP_056397199.1      --------------------------------------------- 609
GCF_001758545.1|WP_071849985.1      KTGIVMLANKFYPGAARIEAAYSILSQLEQRRQ------------ 619
GCF_001425265.1|WP_056397197.1      KAGIVMLSNRSFSGAQRVSAGFEVLSRVAPAGPAVAPAAQSAAAN 621
GCF_900129765.1|WP_073220251.1      KVGIVILANRVLPGDQRVRAAYGLLNQLGPDAP------------ 573
sp|P12287|BLAR_BACLI                --------------------------------------------- 601
```

pepwindowall figures

Hydrophobicity profile of BlaR from *Bacillus licheniformis* (sp|P12287|BLAR_BACLI).

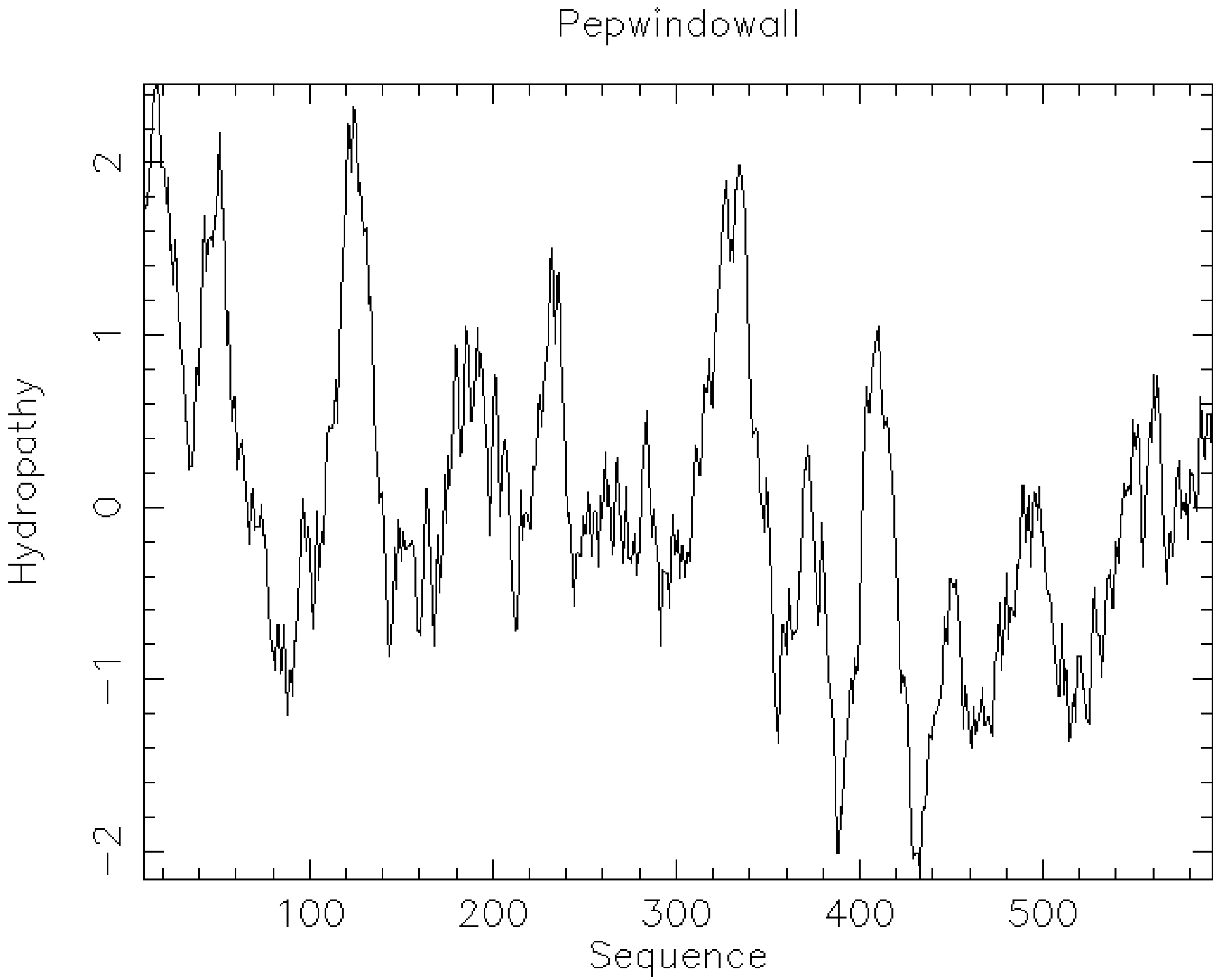

Hydrophobicity profiles of the 14 unaligned long sequences (without the three class C/class D fusion proteins).

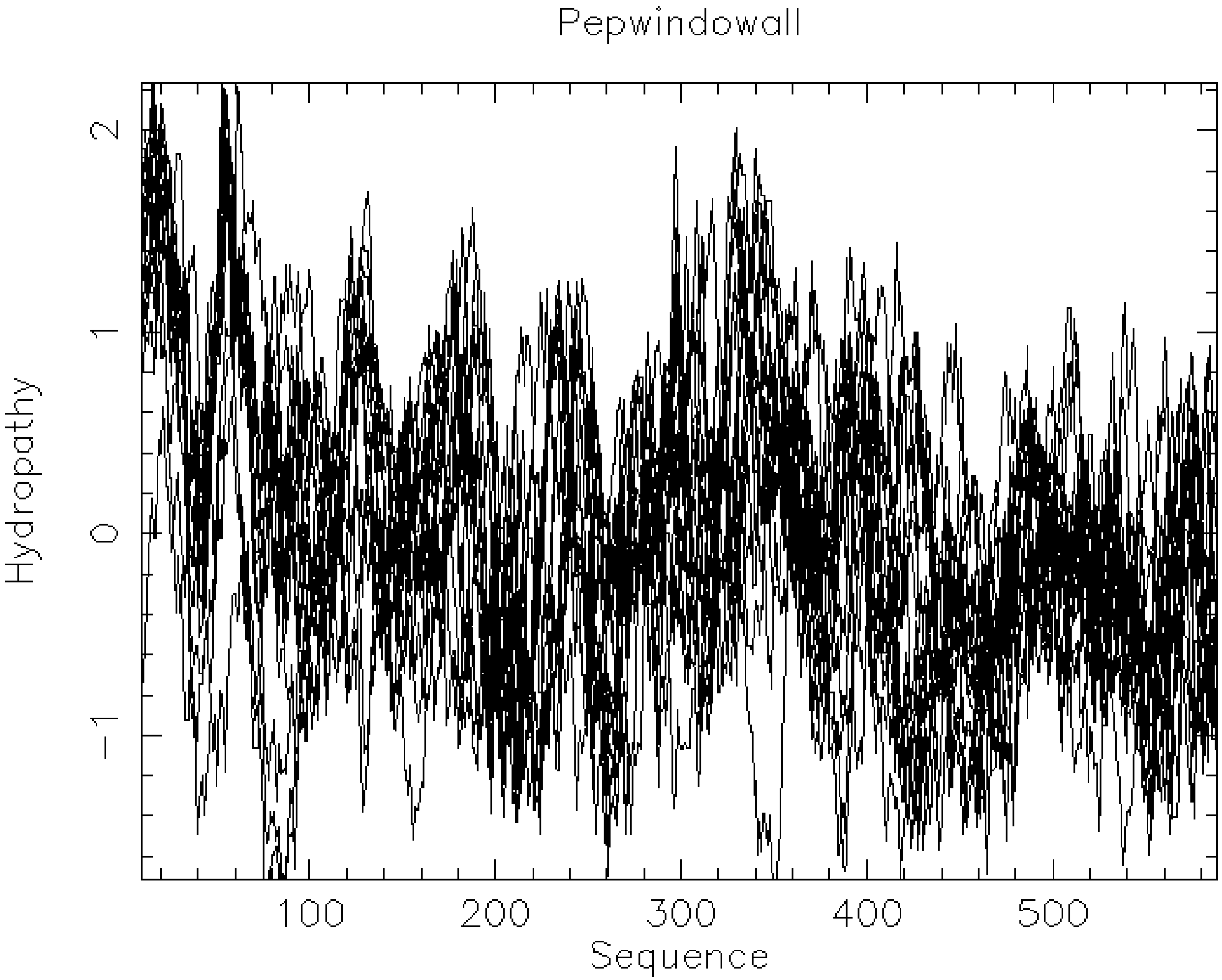

Hydrophobicity profiles of the 14 long sequences (without the three class C/class D fusion proteins) aligned with ClustalO.

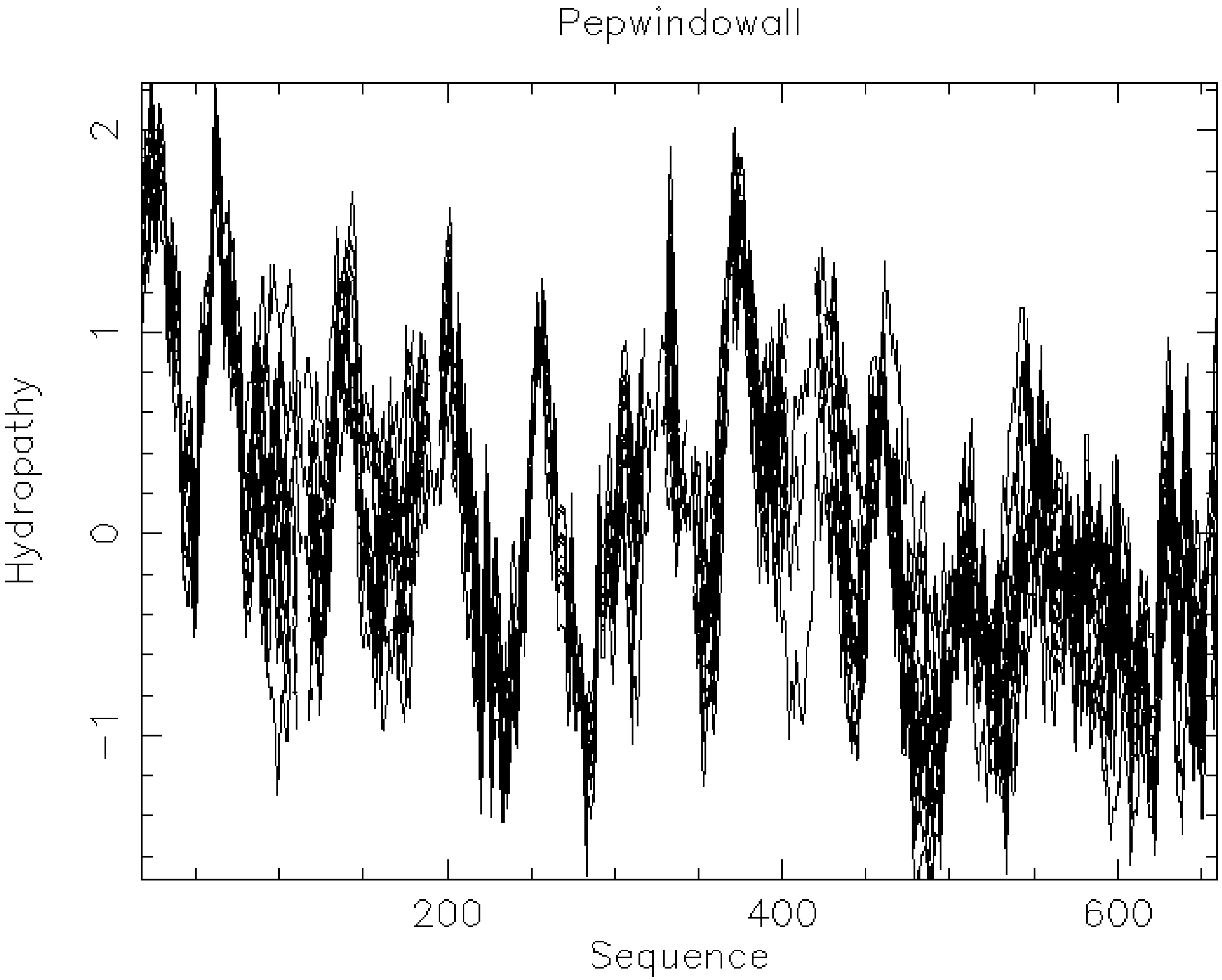

Supplement: SUPPLEMENTAL FILE 1 — Supplemental material. Download SPECTRUM00315-22_Supp_1_seq7.pdf, PDF file, 2.4 MB [file spectrum00315-22_supp_1_seq7.pdf]
